# Gut microbiota in two recently diverged passerine species: evaluating the effects of species identity, habitat use and geographic distance

**DOI:** 10.1186/s12862-021-01773-1

**Published:** 2021-03-10

**Authors:** Camille Sottas, Lucie Schmiedová, Jakub Kreisinger, Tomáš Albrecht, Jiří Reif, Tomasz S. Osiejuk, Radka Reifová

**Affiliations:** 1 Department of Zoology, Faculty of Science, Charles University, Viničná 7, 128 44 Prague, Czech Republic; 2 Institute of Vertebrate Biology, Czech Academy of Sciences, Květná 8, Brno, 603 65 Czech Republic; 3 Faculty of Science, Institute for Environmental Studies, Charles University, Prague, Czech Republic; 4 Department of Zoology and Laboratory of Ornithology, Faculty of Science, Palacky University, Olomouc, Czech Republic; 5 Department of Behavioural Ecology, Institute of Environmental Biology, Faculty of Biology, Adam Mickiewicz University, Poznań, Poland

**Keywords:** Gut microbiome, Reproductive isolation, Diet, Habitat use, Passerines, *Luscinia*

## Abstract

**Background:**

It has been proposed that divergence in the gut microbiota composition between incipient species could contribute to their reproductive isolation. Nevertheless, empirical evidence for the role of gut microbiota in speciation is scarce. Moreover, it is still largely unknown to what extent closely related species in the early stages of speciation differ in their gut microbiota composition, especially in non-mammalian taxa, and which factors drive the divergence. Here we analysed the gut microbiota in two closely related passerine species, the common nightingale (*Luscinia megarhynchos*) and the thrush nightingale (*Luscinia luscinia*). The ranges of these two species overlap in a secondary contact zone, where both species occasionally hybridize and where interspecific competition has resulted in habitat use differentiation.

**Results:**

We analysed the gut microbiota from the proximal, middle and distal part of the small intestine in both sympatric and allopatric populations of the two nightingale species using sequencing of bacterial 16S rRNA. We found small but significant differences in the microbiota composition among the three gut sections. However, the gut microbiota composition in the two nightingale species did not differ significantly between either sympatric or allopatric populations. Most of the observed variation in the gut microbiota composition was explained by inter-individual differences.

**Conclusions:**

To our knowledge, this is the first attempt to assess the potential role of the gut microbiota in bird speciation. Our results suggest that neither habitat use, nor geographical distance, nor species identity have strong influence on the nightingale gut microbiota composition. This suggests that changes in the gut microbiota composition are unlikely to contribute to reproductive isolation in these passerine birds.

**Supplementary Information:**

The online version contains supplementary material available at 10.1186/s12862-021-01773-1.

## Background

Vertebrates harbour taxonomically and functionally diverse microbial communities in their intestines, referred to as the gut microbiota [1, 2]. It has been shown that the composition of the gut microbiota can have profound effects on the host’s physiology and morphology, as well as behaviour [3–8]. Moreover, between-species divergence in the gut microbiota composition could play a role in the establishment of reproductive isolation between species and thus in generating species diversity [6, 9, 10]. Despite recent intensive research on variation in the gut microbiota composition within and between vertebrate species [1, 11–14] the factors that generate the gut microbial diversity are still not sufficiently understood, especially in non-mammalian taxa. Additionally, it is largelly unknown how often closely related species differ in the gut microbiota composition and thus how widespread the effect of the gut microbiota in speciation.

Variation in the gut microbiota composition may arise due to multiple factors including differences in the host’s diet [15–18], habitat [19–21] or geographical range [22–24]. In addition, host genes involved in the management of the gut microbiota can play important roles in structuring gut microbial communities [25–27]. All these factors as well as a stable and long-lasting transfer of the gut microbiota from parents to progeny may generate divergence in the gut microbiota composition between species. However, the importance of specific factors in shaping gut microbiota diversity seems to differ among different vertebrate lineages [28].

Between-species divergence in the gut microbiota composition can contribute to the origin of reproductive isolation by multiple ways. First, host-associated microbiota may be involved in assortative mating and thus the establishment of pre-mating reproductive barriers [6, 9]. Furthermore, interactions between the host genome and the microbiome, between different microbes of the same metagenome, or between different host's genes involved in the management of microbial communities can be disrupted in hybrids [13]. This can cause gut microbiota dysbiosis in hybrid individuals, which can reduce their fitness and contribute to postzygotic isolation [10, 12, 13]. 

Here we studied the gut microbiota variation in two closely related passerine bird species, the common nightingale (*Luscinia megarhynchos*) and the thrush nightingale (*Luscinia luscinia*). The two species diverged approximately 1.8 Mya [29] and their breeding areas currently overlap in a secondary contact zone spanning across Europe [30], where they occasionally hybridize. Both species are migratory and differ in their wintering grounds in sub-Saharan Africa [31]. They both preferentially occupy dense shrubby vegetation (often close to water bodies) and feed mostly on insects [31, 32]. In allopatric regions they inhabit the same habitats, while in the sympatric region their habitat use and diet have partially differentiated, presumably to reduce interspecific competition [32–34]. Common nightingales in sympatry occur more frequently in dry habitats and feed mostly on Coleoptera, whereas thrush nightingales in sympatry prefer wet habitats and feed more often on Diptera [32]. Given that the gut microbiota composition can rapidly shift depending on habitat and prevailing diet [20, 35], the gut microbiota may have differentiated between the two nightingale species in sympatry.

Rarely-occurring interspecific hybrids between the common nightingale and thrush nightingale are viable, but their relative fitness compared to the parental species has not yet been evaluated thoroughly. Nevertheless, it is known that following Haldane’s rule, F_1_ hybrid females are sterile while F_1_ hybrid males are fertile [36–38]. It has been also documented that backcross hybrids are rarely present in the sympatric population [39] and that gene flow can occur between the two species [29, 40].

To elucidate the factors shaping the gut microbiota variation in nightingales, we analysed the gut microbiota profiles in sympatric and allopatric populations of both species using high-throughput sequencing of bacterial 16S rRNA. Unlike most studies on vertebrate gut microbiota based on the analyses of faecal samples as a proxy for intestinal samples, we analysed the microbiota along the whole small intestine to obtain a more complex view of the gut microbiota composition in the two nightingale species. First, we tested whether the gut microbiota composition differs between the two species and whether there are any bacteria exhibiting host species specificity, which would suggest that the gut microbiota could potentially contribute to the reproductive isolation between the two nightingale species. Second, we compared the level of interspecific differences in the gut microbiota composition in sympatry and in allopatry. A higher divergence in sympatry would imply a stronger effect of habitat use or diet, while a higher divergence in allopatry would indicate a stronger effect of geographical region on the gut microbiota divergence [41, 42]. Similar levels of divergence in sympatry and allopatry would suggest that the divergence in host genes involved in the management of the gut microbiota and/or long-term transfer of the gut microbiota from parents to progeny may cause a divergence of the gut microbiota between the two nightingale species. To our knowledge, this study is the first to focus on the gut microbiota composition in a pair of closely related avian species with incomplete reproductive isolation, and to examine its variation in sympatric and allopatric populations. Our findings could have important implications for understanding the factors affecting variation in the gut microbiota composition in birds and the possible role of gut microbiota divergence in avian speciation.

## Results

We sequenced metagenomic DNA extracted from three sections of the small intestine in 18 individuals of the common nightingale (*Luscinia megarhynchos*, hereafter CN) and 18 individuals of the thrush nightingales (*Luscinia luscinia*, hereafter TN). In both species, half of the individuals came from the sympatric region and half from the allopatric region. The three sections of the small intestine were: (1) the duodenum (the proximal part of the small intestine, hereafter DU), (2) the jejunum (the middle part of the small intestine, hereafter JE), and (3) the ileum (the distal part of the small intestine before caecal protuberances, hereafter IL). In total, 108 samples were sequenced (three gut samples for each of the 36 individuals).

**Fig. 1:**
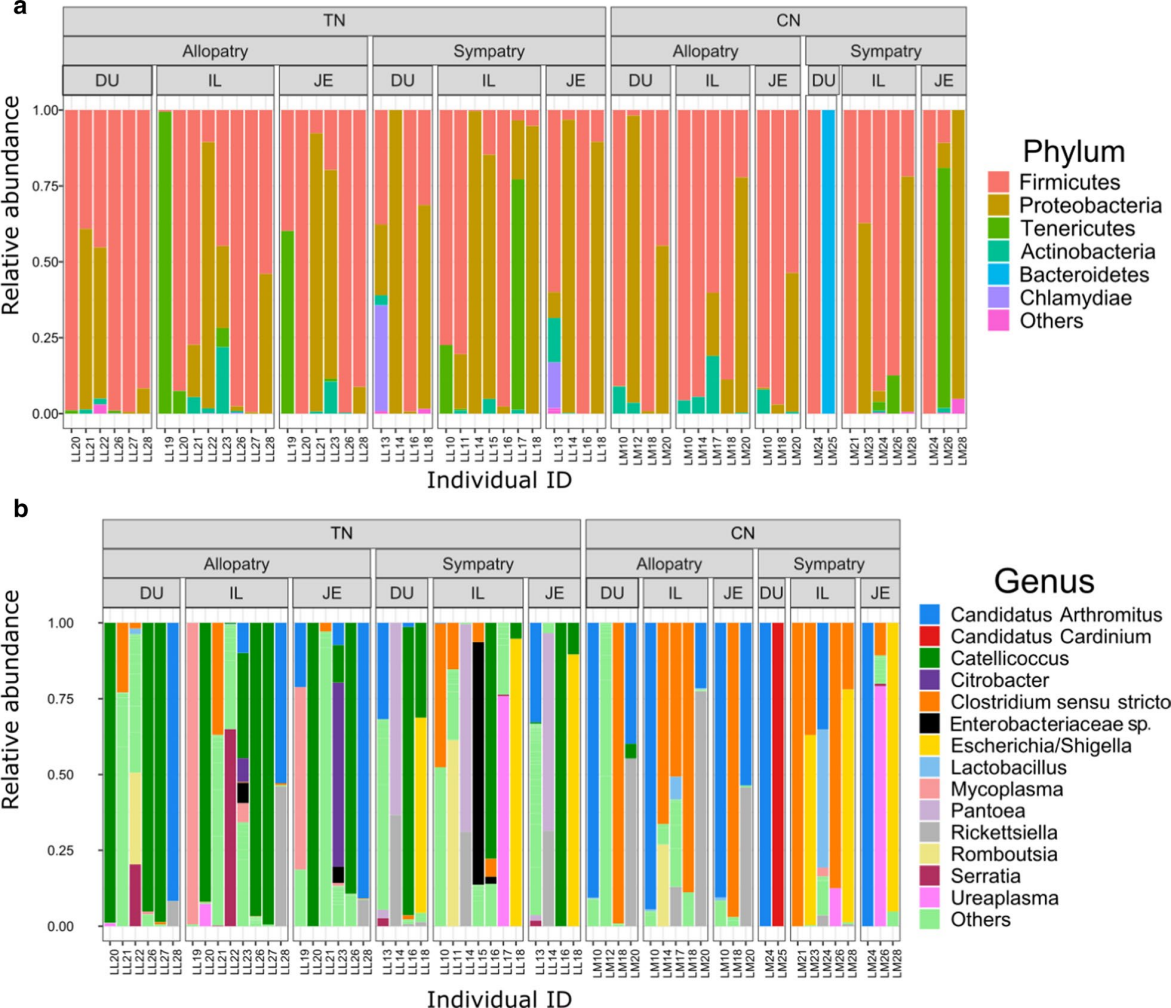
Relative abundances of bacterial phyla (**a**) and genera (**b**) in common nightingale (CN) and thrush nightingale (TN) samples from allopatry and sympatry. The three gut sections (duodenum, DU; jejunum, JE; and ileum, IL) are shown separately. Only the most abundant phyla (relative abundance > 0.5%) and genera (relative abundance > 1%) are represented. Less abundant phyla and genera are included in the category ‘Others’

After the filtering steps, which included (1) removing low-quality sequences, chimeric sequences, sequences not consistently present in both technical duplicates for a given sample, and non-bacterial sequences (including especially reads from coccidia parasites) and (2) excluding samples with less than 1000 reads after all the filtering steps above (see Material and Methods for details), we obtained a final dataset consisting of 57 samples. These included 22 samples from CN (DU = 6, JE = 6 and IL = 10, together representing 12 individuals) and 35 samples from TN (DU = 10, JE = 10 and IL = 15, together representing 16 individuals) (Additional file 1: Table S1). These samples were covered by a total of 276,676 reads. The mean sequencing depth per sample was 4035 (range = 1036–10,261) in CN and 5,369 (range = 1041–14,740) in TN. In total, 272 Operational Taxonomic Units (OTUs) were identified, and the average number of OTUs per sample was 8.33 (range: 1–46). Twelve bacteria phyla and 126 genera were detected in the gut microbiome of the two nightingale species (Fig. 1).

The most common bacterial phyla were Firmicutes (57.95% of relative abundance, dominated by the genera *Catellicoccus, Candidatus Arthromitus* and *Clostridium *sensu* stricto*), Proteobacteria (30.49%, dominated by the genera *Escherichia/Shigella* and *Rickettsiella*), Tenericutes (6.60%, dominated by the genera *Mycoplasma* and *Ureaplasma*), Actinobacteria (1.99%, dominated by the genera *Actinoplanes* and *Kocuria*) and Bacteroidetes (1.76%, dominated by the genus *Candidatus Cardinium*). The relative abundance of all other bacterial phyla was less than 1% (Fig. 1). Regarding the gut sections, Firmicutes and Proteobacteria were the dominant bacterial phyla in all three-gut sections (Fig. 1). The presence of Tenericutes, Bacteroidetes, Actinobacteria and Chlamydia was largely individually specific (Fig. 1).

### Differences in microbial α-diversity among gut sections, between species and regions

**Fig. 2:**
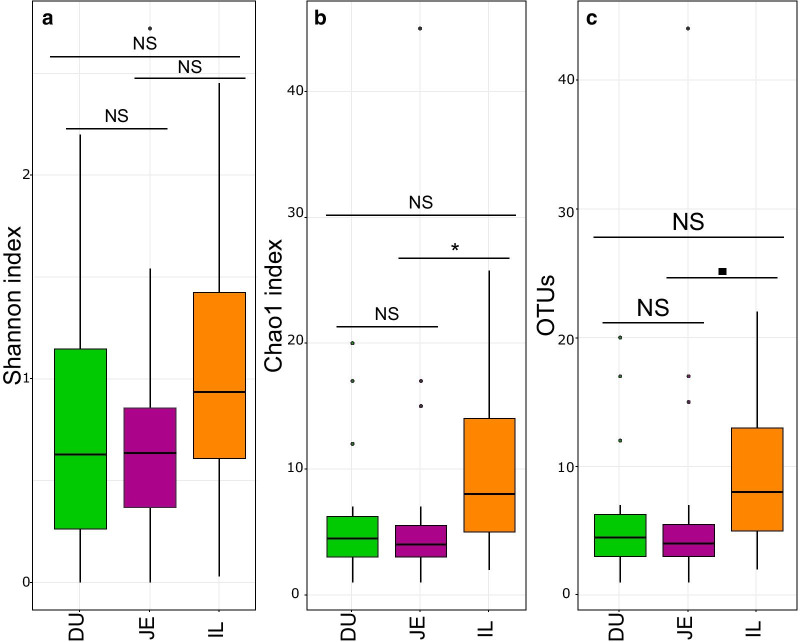
α-diversity of the gut microbiota in the three gut sections across both nightingale species. α-diversity was measured by the Shannon diversity index (**a**), Chao1 diversity index (**b**) and the number of observed OTUs (**c**). To account for uneven sequencing depths, α-diversity indexes were calculated based on the rarefied OTU table (lowest number of reads = 1036). DU stands for duodenum, JE for jejunum and IL for ileum. Results of pairwise post-hoc Tukey tests are shown above horizontal lines (NS p > 0.06, ▪ 0.05 < p < 0.06, *p < 0.05). Box plots depict the median, 1.5 × interquartile range, and range

As estimates of microbial α-diversity, describing the diversity of the microbiome in each sample, we used the Chao1 diversity index (accounting for undetected rare OTUs), the number of observed OTUs and the Shannon diversity index. For all three measures of α-diversity, the microbial diversity was highest in IL (Fig. 2, Table 1 and Additional file 1: Table S2a). We then used linear mixed models (LMMs) to test for the effects of the gut section (i.e. DU, JE, IL), the nightingale species (i.e. CN and TN), geographical region (i.e. allopatry and sympatry) and the species-region interaction on the respective α-diversity indexes. The effect of individual was included as a random effect.

**Table 1:** Effects of the gut section (i.e. duodenum, jejunum and ileum), species (i.e. common nightingale and thrush nightingale), region (i.e. sympatry and allopatry) and the interaction between species and region on α-diversity indexes assessed by linear mixed models

Response variable	Explanatory variable	Chisq	df	p-value
Chao1 index	Gut section	12.050	2	**0.002**
	Species	1.464	1	0.226
	Region	0.102	1	0.749
	Species × region	1.559	1	0.212
Shannon index	Gut section	3.890	2	0.143
	Species	1.239	1	0.265
	Region	0.035	1	0.852
	Species × region	1.625	1	0.202
No. of OTUs	Gut section	11.093	2	**0.004**
	Species	1.512	1	0.219
	Region	0.054	1	0.816
	Species × region	1.545	1	0.214

α-diversity was estimated by Chao1 and Shannon diversity indexes as well as the number of observed OTUs. Individual identity was set as a random effect. Significant p-values are marked in bold

LMMs revealed a significant effect of the gut section on α-diversity for the log-transformed Chao1 index (p = 0.002, Tables 1 and Additional file 1: Table S2a, Fig. 2b) and for the log-transformed number of observed OTUs (p = 0.004, Tables 1 and Additional file 1: Table S2a, Fig. 2a), but not for the Shannon index (Table 1 and Additional file 1: Table S2a, Fig. 2a). Pairwise post-hoc Tukey tests on Chao1 index and the number of observed OTUs showed that α-diversity was significantly higher in IL compared to the JE for the log-Chao1 (p = 0.048). All other pairwise comparisons were, however, insignificant (Fig. 2 and Additional file 1: Table S2b).

**Fig. 3:**
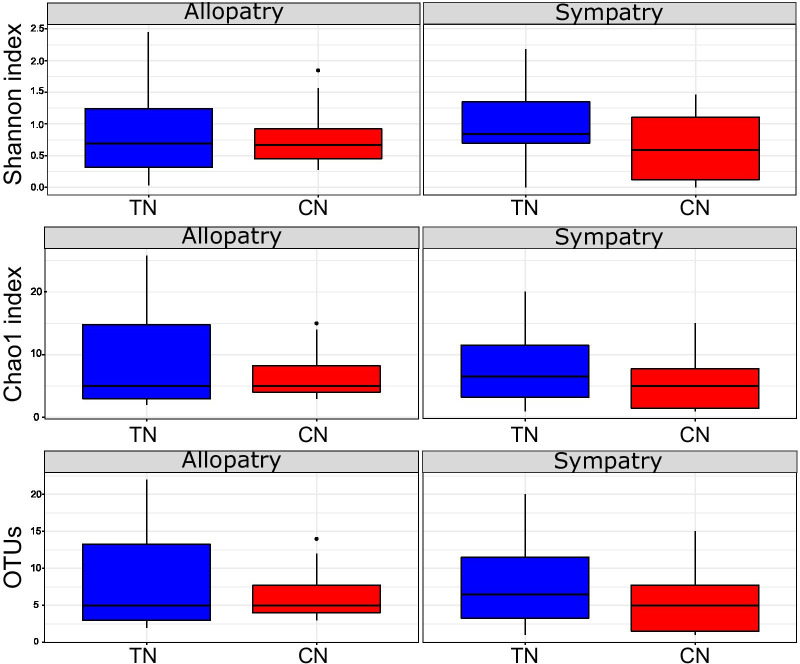
Variation in α-diversity between species and geographical regions. α-diversity was estimated using the Shannon index, Chao1 index and the number of observed OTUs. The thrush nightingale (TN) is represented in blue, while the common nightingale (CN) is represented in red. To account for uneven sequencing depths, α-diversity indexes were calculated based on the rarefied OTU table (the lowest number of reads per sample was 1036). Box plots depict the median, 1.5 × interquartile range, and range

Generally, α-diversity estimates were higher in TN (mean ± standard error (se): Chao1: 9.34 ± 0.37, Shannon: 0.96 ± 0.12, number of observed OTUs: 9.11 ± 1.44) than in CN (mean ± se: Chao1: 6.41 ± 0.30, Shannon: 0.73 ± 0.11, observed OTUs 6.18 ± 0.89). However, α-diversity was higher in TN samples compared to CN samples only in sympatry, not in allopatry (Fig. 3). Nevertheless, when taking into account the inter-individual variability, the effect of the species identity on α-diversity was not significant (LMMs: p > 0.05, Table 1). The effects of the region and the interaction between species identity were also insignificant (LMMs: p > 0.05, Table 1).

### Differences in microbial composition (β-diversity) among gut sections

As measures of microbial composition dissimilarity between samples (β-diversity), we calculated two types of distances: the binary Jaccard distance and the Bray–Curtis distance. The binary Jaccard distance accounts for the presence/absence of OTUs and is thus more sensitive to gut-microbiota changes driven by rare OTUs. The Bray–Curtis distance accounts for differences in the OTUs’ relative abundance and is thus less sensitive to rare OTUs.

**Fig. 4:**
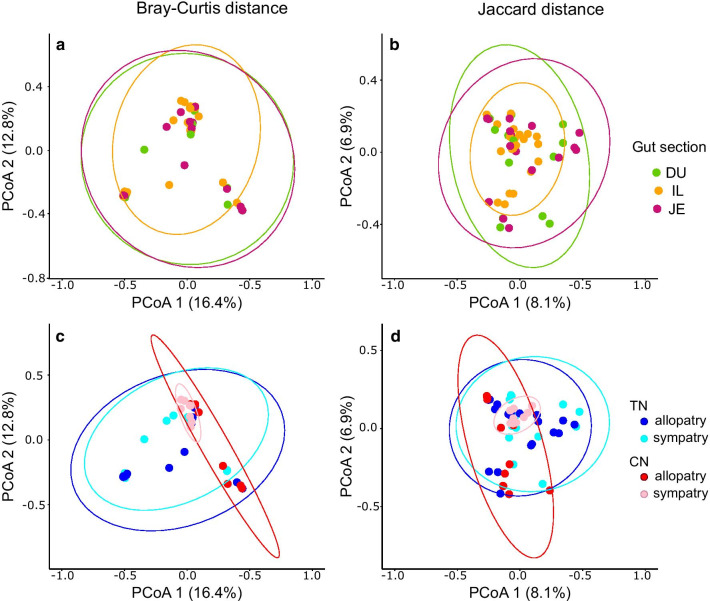
Principal coordinate analyses (PCoA) based on Bray–Curtis (**a**, **c**) and Jaccard (**b**, **d**) distances comparing microbial communities among the three-gut sections (**a**, **b**) and between the two nightingale species (**c**, **d**). DU stands for duodenum, JE for jejunum and IL for ileum. TN stands for thrush nightingale and CN for common nightingale. Coloured ellipses represent 95% confidence intervals. The percentage of variability explained by each axis is shown

We detected within-individual correlations in microbial composition among the three gut sections (Mantel test: p < 0.05 for both distances; range of correlation coefficients is 0.91–0.96 for Bray–Curtis distance and 0.32—0.70 for Jaccard distance; Additional file 1: Table S3). The db-RDA analysis revealed significant differences in the microbiota composition among the three-gut sections for the Jaccard distance (F_2, 54_ = 0.775, p = 0.035) but not for the Bray–Curtis distance (F_2, 54_ = 0.658, p = 0.13). However, the variation in the gut microbiota composition explained by differences among gut sections was very low both for the Jaccard distance (adjusted – R^2^ = 0.028) and Bray–Curtis distance (adjusted – R^2^ = 0.024) (Fig. 4a, b).

### Differences in microbial composition between the two species in sympatry and allopatry

The db-RDA showed no significant effect of species identity or region on the gut microbiota composition (Table 2, model complete) although it revealed a weak but significant interaction between species and regions for both distance matrices (Bray–Curtis distance: p = 0.034 and Jaccard distance: p = 0.043, Table 2, model complete). This may suggest either that the species differ in microbial composition only in sympatry or allopatry, or that some differences within the species may exist between allopatric and sympatric regions. We thus tested these possibilities using db-RDA models focusing separately on each of them. However, the models did not reveal any significant differences in microbial composition between the species in sympatry (Bray–Curtis distance: p = 0.055; Jaccard distance p = 0.078; Table 2) nor in allopatry (Bray–Curtis distance: p = 0.071, Table 2; Jaccard distance p = 0.051; Table 2), although some subtle differences may exist both in sympatry and allopatry (Fig. 4c, d). Moreover, we found no significant differences in microbial composition between allopatric and sympatric regions of CN (Bray–Curtis distance: p = 0.098; Jaccard distance: p = 0.096; Table 2) despite TN showing significant differences in the gut microbiota composition between the two regions for the Bray–Curtis distance (p = 0.014; Table 2).

**Table 2:** Db-RDA analyses testing the effects of species identity (common nightingale vs. thrush nightingale), geographical region (sympatry vs. allopatry) and their interaction on the gut microbial composition

	Dataset	Explanatory variable	p-value	Adjusted – R^2^
Bray–Curtis distance	Complete	Species	0.085	0.139
		Region	0.087	
		Species × region	**0.034**	
	Sympatry	Species	0.055	0.088
	Allopatry	Species	0.071	0.090
	CN	Region	0.098	0.158
	TN	Region	**0.014**	0.049
Jaccard distance	Complete	Species	0.076	0.106
		Region	0.096	
		Species × region	**0.043**	
	Sympatry	Species	0.078	0.077
	Allopatry	Species	0.051	0.061
	CN	Region	0.096	0.101
	TN	Region	0.069	0.056

Analyses were performed on both Bray-Curtis and Jaccard distance matrices, which were the response variables. We also ran the db-RDA analyses to test for the effect of species separately in sympatric and allopatric regions and to test for the effect of region separately in each species. The significance of the models was assessed by a permutation-based ANOVA, where individual identity variation was taken into account during the permutation procedure. Significant p-values are marked in bold. CN stands for common nightingale and TN for thrush nightingale

The nested.anova.dbrda function indicated that the variability in the gut microbiota composition explained by species and region was 14% for the Bray–Curtis distance and 11% for the Jaccard distance, while individual identity explained 79% (Bray Curtis distance) and 67% (Jaccard distance) of the variability in gut microbiota composition (see Table 3).

**Table 3:** Nested analysis of variance via distance-based redundancy

		Df	Sum of squares	F	p value	Variability explained (%)
Bray–Curtis distance	Species/region	3	0.063	1.396	0.171	14
	Individual identity	24	0.364	13.611	**0.001**	79
	Residuals	29	0.032			7
Jaccard distance	Species/region	3	0.050	1.266	0.087	11
	Individual identity	24	0.316	3.641	**0.001**	67
	Residuals	29	0.105			22

Bray–Curtis and Jaccard distance matrices were the response variables, while species identity, region and individual identity were explanatory variables. The number of permutations was set at 1000. Significant p-values are marked in bold

Generalized linear mixed models (GLMMs) identified one OTU belonging to *Clostridium *sensu stricto genus (Firmicutes phylum) that was significantly differentially represented in the two nightingale species in sympatry (Additional file 1: Table S4a). This OTU was more abundant in CN samples than in TN samples. No OTU was significantly differentially represented in the two species in allopatry (Additional file 1: Table S4b). The same OTU belonging to *Clostridium *sensu stricto was also differentially represented between sympatric and allopatric regions of CN as well as TN, although in TN the difference was no longer significant after correcting for multiple testing (Additonal file 1: Table S5a, b). For both species, *Clostridium *sensu stricto was more abundant in sympatry than in allopatry (Additional file 1: Table S5).

## Discussion

Microbial communities living in vertebrate gastrointestinal tracts may affect the fitness-related phenotypic traits of their hosts [3, 43], which in turn may induce selection on mechanisms that ensure the acquisition and maintenance of beneficial microbes. This selection pressure often results in long-lasting stable associations between the host and particular gut microbiota species. As different host species can be co-adapted with different gut bacteria, it is commonly assumed that the gut microbiota can be significantly involved in reproductive isolation between species [13]. However, despite intensive research on various aspects of host gut microbiota interactions over the past decades, empirical evidence for the role of gut microbiota in speciation is still limited and comes mainly from invertebrate taxa [14]. In this study, we examined the gut microbiota composition of two recently diverged songbirds, the common nightingale and the thrush nightingale, in their allopatric and sympatric populations. To our knowledge, this is the first attempt to assess the potential role of gut microbiota in bird speciation.

We found no significant differences in the gut microbiota composition between the two nightingale species, with less than 14% of the total gut microbiota variation being attributed to interspecific dissimilarities. Furthermore, differential abundance analyses identified only a single OTU from the genus *Clostridium* with a significantly different representation between the two nightingale species. Nevertheless, this OTU, as well as other highly prevalent OTUs (e.g. *Candidatus Arthromitus*) were detected in both host species, meaning that none of the OTUs exhibited species specificity. Consequently, our results do not provide support for the existence of species-specific gut microbiota components, and it is thus unlikely that the gut microbiota might be involved in reproductive isolation between the two nightingale species.

Generally, evidence for the role of the host’s microbiota in the origin of reproductive isolation is limited. In various arthropod taxa, bacterial endosymbionts are involved in cytoplasmic incompatibilities [44]. In some arthropods, divergence in the gut microbiome between species can contribute to the mortality of hybrid individuals [14]. It is, however, unclear whether this gut microbiota-induced hybrid lethality arises as a consequence of host vs. gut microbiota incompatibilities or incompatibilities among individual microbial species or the host genes involved in the management of the gut microbiota [13]. In Drosophila, the divergence of host-associated microbiota causes assortative mating between different Drosophila lineages [6, 9], with observed changes in mating preferences caused by changes in levels of cuticular hydrocarbon sex pheromones induced by symbiotic bacteria [9]. In vertebrates, there are a few studies showing phylogenetic co-divergences between hosts and particular bacterial species, typically comprising just a limited fraction of their gut microbiota [10, 12, 45]. Nonetheless, a possible contribution of this gut microbiota divergence to the origin of prezygotic or postzygotic reproductive isolation between species has not yet been demonstrated.

Changes in the host’s gut microbiota can be caused by environmental changes, for example by shifts in the host’s diet or habitat [16, 21, 46]. Such ecological niche shifts associated with changes in the gut microbiota could theoretically also strengthen the degree of reproductive isolation between species. Our previous research documented that sympatric populations of common and thrush nightingales in their secondary contact zone exhibited higher divergence in habitat use [33] and bill morphology [39] compared to allopatric populations. This was consistent with observed interspecific differences in the consumed diet in sympatry [32]. We expected that the greater ecological niche divergence in nightingale sympatric populations would be associated with a higher dissimilarity of their gut microbiota in sympatric compared to allopatric populations. Nevertheless, our data did not support this expectation, as interspecific gut microbiota differences were comparable in both sympatric and allopatric populations. This result corresponds to previous research that revealed a surprisingly low effect of diet and other ecological traits on interspecific gut microbiota variation in a set of bird species with much contrasting ecology than the nightingale species studied here [28, 47]. However, the absence of gut microbiota divergence between the two nightingale species in sympatry might also be a result of interspecific gene flow, as the reproductive isolation between the two species is still incomplete[29, 38, 40].

**Fig. 5:**
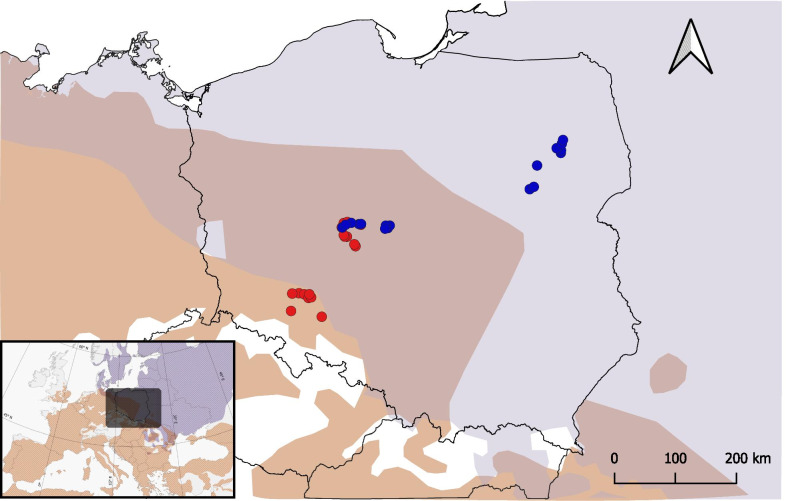
Map of the sampling localities of common nightingales (red dots) and thrush nightingales (blue dots) in Central Europe. Allopatric regions for common nightingales and thrush nightingales are labelled in red and blue, respectively. The sympatric region where both species co-occur is indicated in purple. Species’ ranges are redrawn from [39]

Previous studies have shown a decrease in gut microbiota similarity with increasing geographic distance in various vertebrates [42, 48, 49], suggesting that physical distance could produce barriers to bacterial dispersal. In mammals, species living in allopatry have more dissimilar gut microbiota compositions compared to sympatric species even when controlling for the diet and phylogenetic distance [41]. Our results, showing similar divergence in microbial communities between sympatric and allopatric populations of the two nightingale species, indicate that compared to mammals, geographical distance may not have such a strong effect on the gut microbiota composition in passerine birds. This is generally consistent with previous studies on birds that found no or only weak associations between the gut microbiota composition and geographic distance [47, 49–51]. Nevertheless, as our study area (spanning approximately 600 km; Fig. 5) covered only a part of the two nightingale species geographic range extents, we cannot rule out that some differences in the gut microbiota composition in nightingales may exist over larger geographical distances. The weak effect of geographical distance on bird gut microbiota may be related to the fact that many species, including both our nightingale species, migrate for thousands of km each year to their wintering grounds [31]. Such migrations may be linked with higher dispersal in birds compared to non-migratory vertebrates [52]. In nightingales, natal and breeding dispersal are not known, but our unpublished capture-recapture data on adult birds indicate a high level of fidelity in both species. Males older than one year typically hold the same territories over multiple years. One-year-old males are more dispersive and often settle away from the site of their first breeding, but their movements are generally limited to 15 km, and we have never recorded a translocation over 20 km. Nightingales also show a high degree of migration connectivity [53]. Additionally, a wide variety of habitats and foods utilized during migration itself appears to influence the gut microbiota composition [17], which may also contribute to the weak effect of geographical distance on bird gut microbiota.

While the species identity and geographical region explained only a small amount of the variability of the gut microbiota composition in nightingales (together 14%), individual identity explained more than 79% of the variability. This finding is congruent with other studies on passerine birds, with the gut microbiota typically exhibiting pronounced inter-individual variation [23, 49, 54]. The relatively high inter-individual variability in the gut microbiota composition and the small effect of diet, habitat and species identity on the gut microbiota composition in birds might be related to physiological and morphological adaptations associated with flight, as similar patterns in gut microbiota variation has been observed in bats, which also exhibit reduced intestine sizes and complexity, at least compared to other mammalian clades [28].

The gut microbiota composition in both nightingale species was dominated by the phyla Firmicutes (dominated by the genera *Catellicoccus*, *Candidatus Arthromitus* and *Clostridium *sensu stricto) and Proteobacteria (represented by the genera *Escherichia/Shigella*, *Rickettsiella*, and *Pantoe*a) and was comparable with most passerines studies so far [55–58]. As we analysed the microbiota from three sections of the small intestine, our dataset also provides insight into gut microbiota variations along the digestive tract, which has been rarely studied in birds [51, 55]. We found significant differences in the microbiota composition among the three-gut sections in terms of the bacterial species’ presence/absence, but not in terms of the relative abundances of bacterial species. Nevertheless, the variation in the gut microbiota composition explained by differences among the gut sections was very low (2–3%). The three gut sections also differed in levels of microbial α-diversity, with the ileum—the most distal part of the small intestine—showing higher α-diversity compared to the duodenum and jejunum. The ileum typically maintains a more neutral pH and is responsible for absorption of the remaining products of digestion [59]. We also detected significant within-individual correlations in microbial composition among the three gut sections. Generally, the gut microbiota profile of a particular gut section was more similar to any other gut section from the same individual than to the same gut section from a different individual, suggesting a considerable homogeneity in gut microbiota contents along the nightingale small intestine.

## Conclusion

Our results suggest that neither the species identity, nor habitat, nor geographic distances have significant effects on the gut microbiota composition in the two nightingale species studied here. Instead, individual identity explains most of the observed variation in the gut microbiota composition. Our results are generally consistent with other studies in birds (e.g. [28]), and suggest that ecological factors, including diet and habitat, as well as geographical range do not have a strong influence on the avian gut microbiota composition. Altogether, this indicates that differences in gut microbiota in recently diverged bird species, especially if they are still connected by gene flow, might be usually too small to contribute to the origin of reproductive isolation. Differences in the gut microbiota composition between phylogenetically more distant avian species might arise at later stages of divergence, mostly as a consequence of the long-term independent evolution of species rather than the cause of speciation.

## Methods

### Study area and sampling

The sampling of common nightingales (*Luscinia megarhynchos*) and thrush nightingales (*Luscinia luscinia*) was carried out in Central Europe, in three regions (Fig. 5): an allopatric region for CN (south-western Poland), an allopatric region for TN (north-eastern Poland), and a sympatric region (central Poland) where the ranges of both species overlap and the species often locally co-occur [33]. The allopatric region of CN was close to the sympatric region (Fig. 5); however, according to the Polish Breeding Bird Census data analysed in [33] as well as according to our long-term field observations, no TN individuals were recorded breeding in this area. Moreover, as the south-western edge of TN’s breeding range moved north-east recently (our unpublished observations), at the time of our sampling, CNs allopatric localities were not less than 100 km from the nearest breeding occurrence of TN. Both nightingale species were sampled in May 2018 at the beginning of the breeding season when territories were already established. Only male birds were caught using a mist net with a luring tape. We captured 9 CN and 9 TN males from allopatric regions and 9 CN and 9 TN males from the sympatric region. A list of the sampled birds, including their dates of sampling and GPS coordinates, is provided in Additional file 1: Table S1.

The birds were euthanized by standard cervical dislocation. Dissections started immediately; we removed the entire gastrointestinal tract from the body cavity, and gently separated intestines from the stomach. The whole gut tissue was then placed in a sterilized plastic tube (30 mL) with 99% ethanol, deep-frozen in liquid nitrogen and stored at − 80 °C until DNA extraction. The whole dissection procedure, starting with the euthanasia of the bird and finishing with the gut tissue being stored, did not exceed 8 min. All instruments used to dissect the birds (scissors, lancets) were repeatedly flame-sterilized to prevent cross-individual bacterial contamination of samples. The work with animals was approved by the General Directorate for Environmental Protection, Poland (permission no. DZP-WG.6401.03.123.2017.dl.3).

### DNA extraction from the gut and 16S rRNA sequencing

From each individual’s gut, we dissected three samples (each ca. 0.5 cm long) from the small intestine using sterilized dissection tools. These sections were located in: (1) the duodenum (sampled from the proximal part of the small intestine), (2) the jejunum (sampled from the middle part of the small intestine) and (3) the ileum (the distal part of the small intestine before caecal protuberances). As the passerine colon is very short [11], we were unable to consistently dissect this gut part from all the intestine samples and thus the colon was not analysed in this study. Metagenomic DNA from each sample was extracted using the PowerSoil DNA isolation kit (MO BIO Laboratories Inc., USA). Both sample preparation and DNA extractions took place in a laminar flow cabinet. Sequencing libraries were prepared using a two-step PCR approach. The V3–V4 hypervariable region of bacterial 16S rRNA was amplified using universal primers S-D-Bact-0341-b-S-17 (CCTACGGGNGGCWGCAG) and S-D-Bact-0785-a-A-21 (GACTACHVGGGTATCTAATCC, [60]). Both forward and reverse primers were flanked by oligonucleotides compatible with Nextera adaptors (Illumina, USA). For the first PCR round, 5 μl of KAPA HIFI Hot Start Ready Mix (Kapa Biosystems, USA), 0.2 μM of each primer and 4.6 μl of DNA template were used (final reaction volume = 10 μl). PCR conditions were as follows: initial denaturation at 95 °C for 3 min followed by 30 cycles of 95 °C (30 s), 55 °C (30 s) and 72 °C (30 s), and a final extension at 72 °C (5 min). Dual-indexed Nextera sequencing adaptors were appended to the resulting PCR products during the second PCR. The second PCR reaction consisted of 10 μl of KAPA HIFI Hot Start Ready Mix, 5 μl of H_2_O, 2 μM of each primer and 1 μl of PCR product from the first PCR (final reaction volume = 20 μl) and the PCR program ran for 12 cycles with conditions being the same as during the first PCR. Products from the second PCR round were quantified by GenoSoft software (VWR International, Belgium) based on band intensities after electrophoresis on a 1.5% agarose gel, and mixed at equimolar concentration. The final library was cleaned up using SPRIselect beads (Beckman Coulter Life Sciences, USA). Products of desired size (520–750 bp) were extracted by PipinPrep (Sage Science Inc., USA) and sequenced on an Illumina Miseq (v3 kit, 300 bp paired-end reads). Technical PCR duplicates were sequenced for all individual DNA samples.

### Bioinformatic processing of the sequence data and identification of microbial taxa

Samples were demultiplexed and primers were trimmed by skewer software [61]. Using *dada2* [62], we filtered out low-quality sequences (expected number of errors per read less than 1), denoised the quality-filtered fastq files and constructed an abundance matrix representing reads counts for individual haplotypes (Operational Taxonomic Units, OTUs) in each sample. Using uchime [63] and the gold.fna database (available at https://drive5.com/uchime/gold.fa), we identified chimeric sequences and removed them from the abundance matrix. Taxonomic assignation of haplotypes was conducted by the RDP classifier (80% confidence threshold [64]) and Silva reference database (v 132 [65]).

A large number of sequences from coccidian protozoa, an intracellular parasite present in the intestinal tract of vertebrates provoking Coccidiosis disease [66], were identified in TN samples (43% of the total number of reads) and in CN samples (38% of the total number of reads). These OTUs belonged to the genera *Eimeria* and *Neospora* (phylum: Apicomplexa). We removed all coccidian and other non-bacterial OTUs from the dataset. Furthermore, to eliminate PCR or sequencing artefacts that were not corrected by dada2, we removed all OTUs that were not consistently present in both technical duplicates for a given sample. Read counts for remaining OTUs were subsequently merged for the purpose of all later analyses. Finally, samples with less than 1,000 sequences after all the above filtering steps were discarded. In total, 19 samples from TN and 32 samples from CN were removed.

### Statistical analyses

All statistical analyses were done using packages running under R Statistical Software version 3.4.3 (R Core Team 2015). To account for uneven sequencing depth among samples, a rarefied OTU table (n = 1,036 sequences per sample, which corresponds to the minimal per-sample sequencing depth) was used in all analyses, if not stated otherwise.

### Estimation and comparison of microbial α-diversity

The three α-diversity estimates, including the Chao1 diversity index, the number of observed OTUs and the Shannon diversity index, were calculated using the phyloseq package [67]. LMMs testing the effects of gut section, nightingale species and geographical region on the respective α-diversity indexes were performed in the package lme4 [68]. To account for statistical non-independence (due to sampling of three gut sections for each individual), the effect of individual was included as a random effect. Differences between the gut sections were assessed based on Tukey post-hoc comparisons.

### Dissimilarity of microbial composition (β-diversity) between samples

Two types of distances, the binary Jaccard distance and the Bray-Curtis distance, were calculated as measures of microbial composition dissimilarity between samples (β-diversity) using the vegan package [69].We used a Principal Coordinates Analysis (PCoA) based on the two distance matrices to visualize the differences in microbial composition among the three gut sections across both species. Associations between gut-microbiota composition and gut section were assessed by distance-based redundancy analyses (db-RDAs [70]) with the distance matrix as a response variable and the gut section identity (i.e. DU, JE and IL) as explanatory variables. The significance was assessed by a permutation-based ANOVA, with individual identity being considered as a block (i.e. ‘strata’) for permutation. Additionally, for individuals where all three-gut sections were available (n = 11, Additional file 1: Table S1), within-individual correlations of the microbial composition among the three-gut sections was evaluated using a Mantel’s test (R package ‘ade4’ [71]).

The effects of species identity (i.e. CN and TN) and region (i.e. sympatry and allopatry) on the gut-microbiota composition were assessed via db-RDA. The distance matrix was included as a response variable while nightingale species identity, region and their interaction were included as explanatory variables. The significance of explanatory variables in db-RDAs was assessed by a permutation-based ANOVA. In contrast to the analysis of the gut section, here explanatory variables associated with each individual (i.e. region, species identity) were reshuffled across blocks of individual‐specific samples during the permutation routine to account for the fact that multiple samples for each individual were analysed. To estimate the proportion of the variability explained by each factor on the gut microbiota composition, we used a nested analysis of variance via distance-based redundancy analysis (nested.anova.dbrda; package BiodiversityR [72]). The distance matrices were the response variable, while species identity, region and individual identity were explanatory variables (1000 permutations). To avoid any potential bias due to all three gut sections not being available for some individuals, we also ran this analysis on the subset of individuals (n=11) for which all three gut sections were available. The results were similar for both datasets and we thus present the results only for the whole dataset.

To identify specific OTUs whose abundances differed between the nightingale species in allopatric and sympatric regions, we used generalized linear mixed models with a negative binomial distribution [73]. These analyses were performed on a subset of six OTUs (comprising 43% of all high quality reads) that were detected in at least five samples across both species and regions. The response variable was entered either as (i) the read counts for OTUs from the allopatric region or (ii) the read counts for OTUs from the sympatric region. The explanatory variable was the species identity, and individual identity was set as a random factor. Log-transformed total number of reads per sample was specified as the model offset. A false discovery rate method [74] was subsequently used to account for false discoveries due to multiple tests conducted on the given set of OTUs.

## Supplementary Information

**Additional file 1: Table S1.** A list of sampled nightingale individuals with information about their species, geographical region, date of sampling and GPS coordinates. **Table S2.** α-diversity in the three gut sections across both nightingale species (a) and pairwise post-hoc Tukey tests of differences in α-diversity between specific gut sections (b) **Table S3.** Within-individual correlations in the microbial composition among the three-gut sections. **Table S4.** Differences in representation of particular OTUs between the two nightingale species in sympatry (a) and in allopatry (b). **Table S5.** Differences in representation of particular OTUs between sympatric and allopatric region in the common nightingale (a) and in the thrush nightingale (b). **Table S6.** Metadata to the individual sequence samples that are available from the European Nucleotide Archive under the study accession number: PRJEB43057.

## Data Availability

Sequencing data are available from the European Nucleotide Archive under the study accession number: PRJEB43057. Metadata to the individual sequence samples are provided in the Supplementary Material Table S6. All other data are attached as a Supplementary Material.

## Abbreviations

CN: Common nightingale
TN: Thrush nightingale
OTU: Operational taxonomic unit
LMM: Linear mixed model
PCoA: Principal coordinates analysis
db-RDA: Distance-based redundancy analyses
DU: Duodenum
JE: Jejunum
IL: Ileum
GLMM: General mixed model
ANOVA: Analysis of variance
PCR: Polymerase chain reaction
