# Supplementary material for: Gut microbiota in two recently diverged passerine species: evaluating the effects of species identity, habitat use and geographic distance
Source: BMC Ecol Evol. 2021 Mar 10;21:41. doi: 10.1186/s12862-021-01773-1 (PMC7948333; doi:10.1186/s12862-021-01773-1)
Supplement: Supplementary file 1 — Additional file 1: Table S1. A list of sampled nightingale individuals with information about their species, geographical region, date of sampling and GPS coordinates. Table S2. α-diversity in the three gut sections across both nightingale species (a) and pairwise post-hoc Tukey tests of differences in α-diversity between specific gut sections (b) Table S3. Within-individual correlations in the microbial composition among the three-gut sections. Table S4. Differences in representation of particular OTUs between the two nightingale species in sympatry (a) and in allopatry (b). Table S5. Differences in representation of particular OTUs between sympatric and allopatric region in the common nightingale (a) and in the thrush nightingale (b). Table S6. Metadata to the individual sequence samples that are available from the European Nucleotide Archive under the study accession number: PRJEB43057. [file 12862_2021_1773_MOESM1_ESM.docx]

Additional material

**Gut microbiota in two recently diverged passerine species: evaluating the effects of species identity, habitat use and geographic distance**

Camille Sottas^1^, Lucie Schmiedová^1^, Jakub Kreisinger^1^, Tomáš Albrecht^1, 2^, Jiří Reif ^3, 4^, Tomasz S. Osiejuk^5^, Radka Reifová^1^

^1^Department of Zoology, Faculty of Science, Charles University, Prague, Czech Republic

^2^Institute of Vertebrate Biology, Czech Academy of Sciences, Květná 8, Brno 603 65, Czech Republic

^3^Faculty of Science, Institute for Environmental Studies, Charles University, Prague, Czech Republic

^4^Department of Zoology and Laboratory of Ornithology, Faculty of Science, Palacky University, Olomouc, Czech Republic

^5^[Department of Behavioural Ecology](https://amu.academia.edu/Departments/Department_of_Behavioural_Ecology/Documents), Institute of Environmental Biology, Faculty of Biology, [Adam Mickiewicz University in Poznań](https://amu.academia.edu/), Poland

## **Table S1**. A list of sampled nightingale individuals with information about their species identity, geographical region, date of sampling and GPS coordinates. For each individual number of sequence reads in the three gut sections (e.g. duodenum (DU), jejunum (JE) and ileum (IL)) are shown. TN stands for thrush nightingale and CN for common nightingale.

| Individual | Species | Region | Date | Latitude | Longitude | Gut section | Before filtering | | After filtering |
| --- | --- | --- | --- | --- | --- | --- | --- | --- | --- |
|  |  |  |  |  |  |  | Total number of reads | Total number of coccidian and other non-bacterial reads | Total number of reads |
| LL_10 | TN | sympatry | 11.5.2018 | 52.149 | 17.672 | DU | 5496 | 5182 | 0 |
|  |  |  |  |  |  | JE | 5252 | 5056 | 0 |
|  |  |  |  |  |  | IL | 9300 | 1128 | 6952 |
| LL_11 | TN | sympatry | 11.5.2018 | 52.195 | 18.037 | DU | 7869 | 7446 | 0 |
|  |  |  |  |  |  | JE | 12801 | 12691 | 0 |
|  |  |  |  |  |  | IL | 6599 | 522 | 5460 |
| LL_12 | TN | sympatry | 11.5.2018 | 52.193 | 18.058 | DU | 4430 | 4326 | 0 |
|  |  |  |  |  |  | JE | 3580 | 3466 | 0 |
|  |  |  |  |  |  | IL | 5414 | 5296 | 0 |
| LL_13 | TN | sympatry | 11.5.2018 | 52.204 | 17.864 | DU | 6356 | 4060 | 1521 |
|  |  |  |  |  |  | JE | 6509 | 507 | 4312 |
|  |  |  |  |  |  | IL | 7594 | 7086 | 0 |
| LL_14 | TN | sympatry | 11.5.2018 | 52.203 | 17.786 | DU | 12443 | 1814 | 8881 |
|  |  |  |  |  |  | JE | 12943 | 535 | 10160 |
|  |  |  |  |  |  | IL | 8870 | 1241 | 6415 |
| LL_15 | TN | sympatry | 12.5.2018 | 52.150 | 18.655 | DU | 7573 | 7474 | 0 |
|  |  |  |  |  |  | JE | 6777 | 6262 | 0 |
|  |  |  |  |  |  | IL | 5405 | 1997 | 2661 |
| LL_16 | TN | sympatry | 12.5.2018 | 52.160 | 18.675 | DU | 7439 | 4949 | 2072 |
|  |  |  |  |  |  | JE | 6161 | 3743 | 2216 |
|  |  |  |  |  |  | IL | 12748 | 254 | 10900 |
| LL_17 | TN | sympatry | 12.5.2018 | 52.17 | 18.664 | DU | 4481 | 4300 | 0 |
|  |  |  |  |  |  | JE | 8789 | 8317 | 0 |
|  |  |  |  |  |  | IL | 8900 | 6232 | 2244 |
| LL_18 | TN | sympatry | 12.5.2018 | 52.143 | 18.615 | DU | 6459 | 4753 | 1507 |
|  |  |  |  |  |  | JE | 10196 | 8420 | 1441 |
|  |  |  |  |  |  | IL | 12961 | 6052 | 6135 |
| LL_19 | TN | allopatry | 14.5.2018 | 52.658 | 21.738 | DU | 5008 | 4491 | 0 |
|  |  |  |  |  |  | JE | 6969 | 5724 | 1041 |
|  |  |  |  |  |  | IL | 12974 | 1577 | 9318 |
| LL_20 | TN | allopatry | 14.5.2018 | 52.689 | 21.829 | DU | 8807 | 1038 | 7287 |
|  |  |  |  |  |  | JE | 16571 | 2398 | 13320 |
|  |  |  |  |  |  | IL | 16385 | 1072 | 14325 |
| LL_21 | TN | allopatry | 14.5.2018 | 52.970 | 21.899 | DU | 6507 | 4325 | 2028 |
|  |  |  |  |  |  | JE | 7579 | 4879 | 2297 |
|  |  |  |  |  |  | IL | 10767 | 1933 | 7576 |
| LL_22 | TN | allopatry | 15.5.2018 | 53.134 | 22.416 | DU | 7911 | 4732 | 1624 |
|  |  |  |  |  |  | JE | 6748 | 6697 | 0 |
|  |  |  |  |  |  | IL | 9398 | 2075 | 5670 |
| LL_23 | TN | allopatry | 15.5.2018 | 53.167 | 22.421 | DU | 4982 | 3996 | 0 |
|  |  |  |  |  |  | JE | 8219 | 4046 | 3312 |
|  |  |  |  |  |  | IL | 6636 | 3863 | 2017 |
| LL_25 | TN | allopatry | 15.5.2018 | 53.195 | 22.325 | DU | 3272 | 3211 | 0 |
|  |  |  |  |  |  | JE | 5351 | 4095 | 0 |
|  |  |  |  |  |  | IL | 5269 | 5209 | 0 |
| LL_26 | TN | allopatry | 15.5.2018 | 53.196 | 22.325 | DU | 19035 | 3182 | 14740 |
|  |  |  |  |  |  | JE | 7437 | 2568 | 4465 |
|  |  |  |  |  |  | IL | 10580 | 2856 | 7070 |
| LL_27 | TN | allopatry | 15.5.2018 | 53.254 | 22.437 | DU | 4307 | 1589 | 2549 |
|  |  |  |  |  |  | JE | 4882 | 4160 | 0 |
|  |  |  |  |  |  | IL | 5328 | 785 | 4297 |
| LL_28 | TN | allopatry | 15.5.2018 | 53.304 | 22.463 | DU | 5044 | 365 | 4290 |
|  |  |  |  |  |  | JE | 4110 | 1576 | 2357 |
|  |  |  |  |  |  | IL | 6825 | 594 | 5454 |
| LM_10 | CN | allopatry | 6.5.2018 | 51.020 | 16.559 | DU | 4878 | 525 | 3921 |
|  |  |  |  |  |  | JE | 3919 | 483 | 3180 |
|  |  |  |  |  |  | IL | 4148 | 1614 | 2046 |
| LM_11 | CN | allopatry | 6.5.2018 | 50.942 | 17.227 | DU | 2713 | 2486 | 0 |
|  |  |  |  |  |  | JE | 2713 | 2414 | 0 |
|  |  |  |  |  |  | IL | 2052 | 2025 | 0 |
| LM_12 | CN | allopatry | 6.5.2018 | 50.9256 | 17.227 | DU | 5105 | 419 | 2303 |
|  |  |  |  |  |  | JE | 403 | 121 | 0 |
|  |  |  |  |  |  | IL | 2481 | 1122 | 0 |
| LM_13 | CN | allopatry | 7.5.2018 | 51.264 | 16.730 | DU | 7773 | 7717 | 0 |
|  |  |  |  |  |  | JE | 14063 | 13973 | 0 |
|  |  |  |  |  |  | IL | 5444 | 5401 | 0 |
| LM_14 | CN | allopatry | 7.5.2018 | 51.252 | 16.598 | DU | 4363 | 4324 | 0 |
|  |  |  |  |  |  | JE | 3948 | 601 | 0 |
|  |  |  |  |  |  | IL | 4466 | 258 | 1199 |
| LM_17 | CN | allopatry | 7.5.2018 | 51.249 | 16.846 | DU | 4074 | 3019 | 0 |
|  |  |  |  |  |  | JE | 3234 | 3223 | 0 |
|  |  |  |  |  |  | IL | 3741 | 419 | 1979 |
| LM_18 | CN | allopatry | 7.5.2018 | 51.204 | 16.965 | DU | 4815 | 206 | 4339 |
|  |  |  |  |  |  | JE | 4119 | 928 | 2928 |
|  |  |  |  |  |  | IL | 12961 | 6052 | 4839 |
| LM_19 | CN | allopatry | 7.5.2018 | 51.201 | 16.968 | DU | 2274 | 1736 | 0 |
|  |  |  |  |  |  | JE | 4197 | 4123 | 0 |
|  |  |  |  |  |  | IL | 1174 | 1035 | 0 |
| LM_20 | CN | allopatry | 7.5.2018 | 51.248 | 16.902 | DU | 4917 | 1220 | 3374 |
|  |  |  |  |  |  | JE | 5246 | 612 | 4186 |
|  |  |  |  |  |  | IL | 10331 | 417 | 8720 |
| LM_21 | CN | sympatry | 10.5.2018 | 51.899 | 17.961 | DU | 1287 | 414 | 0 |
|  |  |  |  |  |  | JE | 2490 | 2113 | 0 |
|  |  |  |  |  |  | IL | 3949 | 74 | 3590 |
| LM_22 | CN | sympatry | 10.5.2018 | 51.924 | 17.935 | DU | 536 | 190 | 0 |
|  |  |  |  |  |  | JE | 2224 | 2098 | 0 |
|  |  |  |  |  |  | IL | 2494 | 2221 | 0 |
| LM_23 | CN | sympatry | 10.5.2018 | 52.0268 | 17.775 | DU | 1869 | 876 | 0 |
|  |  |  |  |  |  | JE | 5585 | 4860 | 0 |
|  |  |  |  |  |  | IL | 12247 | 259 | 10261 |
| LM_24 | CN | sympatry | 10.5.2018 | 52.028 | 17.724 | DU | 4283 | 345 | 3740 |
|  |  |  |  |  |  | JE | 4889 | 715 | 3943 |
|  |  |  |  |  |  | IL | 9068 | 502 | 7027 |
| LM_25 | CN | sympatry | 10.5.2018 | 52.217 | 17.751 | DU | 4753 | 2176 | 237 |
|  |  |  |  |  |  | JE | 1074 | 974 | 0 |
|  |  |  |  |  |  | IL | 5067 | 5032 | 0 |
| LM_26 | CN | sympatry | 10.5.2018 | 52.208 | 17.793 | DU | 3294 | 2610 | 0 |
|  |  |  |  |  |  | JE | 8201 | 5384 | 2185 |
|  |  |  |  |  |  | IL | 3726 | 124 | 3379 |
| LM_27 | CN | sympatry | 11.5.2018 | 52.148 | 17.670 | DU | 4566 | 4506 | 0 |
|  |  |  |  |  |  | JE | 4046 | 4011 | 0 |
|  |  |  |  |  |  | IL | 5254 | 5156 | 0 |
| LM_28 | CN | sympatry | 11.5.2018 | 52.189 | 18.056 | DU | 2738 | 2476 | 0 |
|  |  |  |  |  |  | JE | 2478 | 1348 | 1036 |
|  |  |  |  |  |  | IL | 9775 | 239 | 8216 |
| LM_29 | CN | sympatry | 11.5.2018 | 52.048 | 17.704 | DU | 4231 | 4012 | 0 |
|  |  |  |  |  |  | JE | 6785 | 6157 | 0 |
|  |  |  |  |  |  | IL | 6535 | 5708 | 0 |

## **Table S2.** α-diversity in the three gut sections across both nightingale species (a) and pairwise post-hoc Tukey tests of differences in α-diversity between specific gut sections (b) α-diversity was estimated using Shannon and Chao1 diversity indexes and number of observed OTUs. DU stands for duodenum, JE for jejunum, and IL for ileum. Results of post-hoc Tukey tests are shown only for Chao 1 index and no. of OTUs for which gut section had significant effect on α-diversity in the LME models. Mean ± standard error (SE) and p-values are shown. Significant p-values are in bold.

| (a) | DU |  | JE |  | IL |
| --- | --- | --- | --- | --- | --- |
|  | mean ± SE |  | mean ± SE |  | mean ± SE |
| Shannon | 0.77 ±0.18 |  | 0.75 ±0.17 |  | 1.02 ±0.12 |
| Chao1 | 6.25 ±0.04 |  | 7.56 ±0.19 |  | 9.87 ±0.64 |
| OTUs | 6.25 ±1.37 |  | 7.50 ±2.68 |  | 9.40 ±1.06 |

| b) | DU-JE |  | JE-IL |  | IL-DU |
| --- | --- | --- | --- | --- | --- |
|  | p-value |  | p-value |  | p-value |
| Chao1 | 0.994 |  | **0.048** |  | 0.061 |
| OTUs | 0.993 |  | 0.058 |  | 0.076 |

## **Table S3.** Within-individual correlations in the microbial composition among the three-gut sections. Correlations were evaluated using a Mantel test on Bray-Curtis and Jaccard distances. The test was carried out on individuals, for which all three-gut sections were available (n = 11). DU stands for duodenum, JE for jejunum and IL ileum. Correlation coefficients and p-values are shown.

|  | | Bray-Curtis distance | |  | Jaccard distance | |
| --- | --- | --- | --- | --- | --- | --- |
|  | correlation coefficient | | p-value |  | correlation coefficient | p-value |
| DU-JE | 0.96 | | 1.00e-04 |  | 0.42 | 0.02 |
| JE-IL | 0.91 | | 1.00e-04 |  | 0.70 | 1.00e-04 |
| IL-DU | 0.93 | | 1.00e-04 |  | 0.32 | 0.04 |

## **Table S4.** Differences in representation of particular OTUs between the two nightingale species in sympatry (a) and in allopatry (b). Significance of differences were tested by generalized linear mixed models. Significant differences after correction for multiple testing (false discovery rate – FDR) are in bold. Only OTUs identified in more than five nightingale individuals across both species were used in this analysis.

| (a) |  |  |  |  |  |  |  |  |  | |  | |
| --- | --- | --- | --- | --- | --- | --- | --- | --- | --- | --- | --- | --- |
|  |  |  |  |  |  |  |  |  | common nightingale | | thrush nightingale | |
| OTU | Phylum | Class | Order | Family | Genus | z | p | FDR | No. of individuals^1^ | Relative abundance (%)^2^ | No. of individuals^1^ | Relative abundance (%)^2^ |
| OTU  15935 | Firmicutes | Clostridia | Clostridiales | Clostridiaceae_1 | *Clostrdium*  *sensu stricto* | 6.413 | **<0.001** | **<0.001** | 5 | 25.35 | 6 | 2.58 |
| OTU  12053 | Firmicutes | Clostridia | Clostridiales | Lachnospiraceae | *Tyzzerella_3* | -0.001 | 0.999 | 1 | 0 | 0 | 4 | 3.03 |
| OTU  16501 | Firmicutes | Bacilli | Lactobacillales | Enterococcaceae | *Catellicoccus* | -0.002 | 0.998 | 1 | 0 | 0 | 7 | 18.64 |
| OTU  16527 | Firmicutes | Bacilli | Lactobacillales | Carnobacteriaceae | *Carnobacterium* | -0.001 | 0.100 | 1 | 0 | 0 | 2 | 0.18 |
| OTU  22474 | Firmicutes | Clostridia | Clostridiales | Clostridiaceae_1 | *Candidatus*  *Arthromitus* | -0.0001 | 1 | 1 | 0 | 0 | 4 | 2.63 |
| OTU  15903 | Firmicutes | Clostridia | Clostridiales | Clostridiaceae_1 | *Clostrdium_*  *sensu stricto* | NA | NA | NA | 0 | 0 | 0 | 0 |

| (b) |  |  |  |  |  |  |  |  |  | |  | |
| --- | --- | --- | --- | --- | --- | --- | --- | --- | --- | --- | --- | --- |
|  |  |  |  |  |  |  |  |  | common nightingale | | thrush nightingale | |
| OTU | Phylum | Class | Order | Family | Genus | z | p | FDR | No. of individuals^1^ | Relative abundance (%)^2^ | No. of individuals^1^ | Relative abundance (%)^2^ |
| OTU 15935 | Firmicutes | Clostridia | Clostridiales | Clostridiaceae_1 | *Clostridium*  *sensu stricto* | -0.261 | 0.794 | 0.999 | 1 | 1.84 | 3 | 0.06 |
| OTU 12053 | Firmicutes | Clostridia | Clostridiales | Lachnospiraceae | *Tyzzerella_3* | -0.001 | 0.999 | 0.999 | 0 | 0 | 3 | 0.97 |
| OTU 16501 | Firmicutes | Bacilli | Lactobacillales | Enterococcaceae | *Catellicoccus* | -2.322 | 0.020 | 0.121 | 1 | 0.27 | 9 | 40.28 |
| OTU 16527 | Firmicutes | Bacilli | Lactobacillales | Carnobacteriaceae | *Carnobacterium* | -0.089 | 0.929 | 0.999 | 1 | 0.10 | 4 | 0.85 |
| OTU 22474 | Firmicutes | Clostridia | Clostridiales | Clostridiaceae_1 | *Candidatus*  *Arthromitus* | 0.381 | 0.703 | 0.999 | 6 | 31.98 | 5 | 8.33 |
| OTU 15903 | Firmicutes | Clostridia | Clostridiales | Clostridiaceae_1 | *Clostridium*  *sensu stricto* | -0.181 | 0.856 | 0.999 | 4 | 24.73 | 7 | 2.98 |

^1^ The number of nightingale individuals containing a bacterial taxon. ^2^ The proportion of reads for a given OTU.

## **Table S5.** Differences in representation of particular OTUs between sympatric and allopatric region in the common nightingale (a) and in the thrush nightingale (b). Significance of differences was tested by a generalized linear mixed model. Significant differences after correction for multiple testing (false discovery rate – FDR) are in bold. Only OTUs identified in more than five nightingale individuals across both species were used in this analysis.

| (a) |  |  |  |  |  |  |  |  |  |  |  |  |
| --- | --- | --- | --- | --- | --- | --- | --- | --- | --- | --- | --- | --- |
| OTU | Phylum | Class | Order | Family | Genus | z | p.value | FDR | sympatry | | allopatry | |
|  |  |  |  |  |  |  |  |  | No. of  individuals^1^ | Relative abundance (%)^2^ | No. of  individuals^1^ | Relative abundance (%)^2^ |
| OTU  15935 | Firmicutes | Clostridia | Clostridiales | Clostridiaceae_1 | *Clostridium*  *sensu stricto* | 5.583 | **<0.001** | **<0.001** | 5 | 25.35 | 1 | 1.84 |
| OTU  12053 | Firmicutes | Clostridia | Clostridiales | Lachnospiraceae | *Tyzzerella_3* | NA | NA | NA | 0 | 0 | 0 | 0 |
| OTU  16501 | Firmicutes | Bacilli | Lactobacillales | Enterococcaceae | *Catellicoccus* | -0.001 | 0.999 | 0.999 | 0 | 0 | 1 | 0.27 |
| OTU  16527 | Firmicutes | Bacilli | Lactobacillales | Carnobacteriaceae | *Carnobacterium* | -0.010 | 0.992 | 0.999 | 0 | 0 | 1 | 0.10 |
| OTU  22474 | Firmicutes | Clostridia | Clostridiales | Clostridiaceae_1 | *Candidatus*  *Arthromitus* | -0.001 | 0.999 | 0.999 | 0 | 0 | 6 | 31.98 |
| OTU  15903 | Firmicutes | Clostridia | Clostridiales | Clostridiaceae_1 | *Clostridium sensu stricto* | -0.119 | 0.905 | 0.999 | 0 | 0 | 4 | 24.73 |

| (b) |  |  |  |  |  | |  |  | |  |  | |  | | |  |  |
| --- | --- | --- | --- | --- | --- | --- | --- | --- | --- | --- | --- | --- | --- | --- | --- | --- | --- |
| OTU | Phylum | Class | Order | Family | | Genus | | z | p.value | FDR | | sympatry | | | allopatry | | |
|  |  |  |  |  |  |  |  |  |  |  |  | No. of  individuals^1^ | | Relative abundance (%)^2^ | No. of  individuals^1^ | | Relative abundance (%)^2^ |
| OTU  15935 | Firmicutes | Clostridia | Clostridiales | Clostridiaceae_1 | | *Clostridium*  *sensu stricto* | | 2.532 | 0.011 | 0.068 | | 6 | | 2.58 | 3 | | 0.06 |
| OTU  12053 | Firmicutes | Clostridia | Clostridiales | Lachnospiraceae | | *Tyzzerella_3* | | 1.873 | 0.061 | 0.183 | | 4 | | 3.03 | 3 | | 0.97 |
| OTU  16501 | Firmicutes | Bacilli | Lactobacillales | Enterococcaceae | | *Catellicoccus* | | -0.391 | 0.696 | 0.929 | | 7 | | 18.64 | 9 | | 40.28 |
| OTU  16527 | Firmicutes | Bacilli | Lactobacillales | Carnobacteriaceae | | *Carnobacterium* | | 0.287 | 0.774 | 0.929 | | 2 | | 0.18 | 4 | | 0.85 |
| OTU  22474 | Firmicutes | Clostridia | Clostridiales | Clostridiaceae_1 | | *Candidatus*  *Arthromitus* | | 0.485 | 0.628 | 0.929 | | 4 | | 2.63 | 5 | | 8.33 |
| OTU  15903 | Firmicutes | Clostridia | Clostridiales | Clostridiaceae_1 | | *Clostridium sensu stricto* | | -0.002 | 0.999 | 0.999 | | 0 | | 0 | 7 | | 2.98 |

^1^ The number of nightingale individuals containing a bacterial taxon. ^2^ The proportion of reads for a given OTU.

## **Table S6**. Metadata to the individual sequence samples that are available from the European Nucleotide Archive under the study accession number: PRJEB43057. Sample names, nightingale individual identity (ID), species identity (CN stands for common nightingale and TN for thrush nightingale), sex, geographical region, gut section (DU stands for duodenum, JE stands for jejunum and IL stands for and ileum), alias name and accession numbers are shown.

| Sample name | Individual ID | Species | Sex | Region | Gut section | Alias | Accession |
| --- | --- | --- | --- | --- | --- | --- | --- |
| LM19_IL | LM19 | CN | M | allopatry | IL | Nightingale_GUT_MICRO77 | ERS5713386 (SAMEA8026169) |
| LM19_JE | LM19 | CN | M | allopatry | JE | Nightingale_GUT_MICRO78 | ERS5713387 (SAMEA8026170) |
| LM19_DU | LM19 | CN | M | allopatry | DU | Nightingale_GUT_MICRO76 | ERS5713385 (SAMEA8026167) |
| LM22_IL | LM22 | CN | M | sympatry | IL | Nightingale_GUT_MICRO86 | ERS5713395 (SAMEA8026178) |
| LM22_JE | LM22 | CN | M | sympatry | JE | Nightingale_GUT_MICRO87 | ERS5713396 (SAMEA8026179) |
| LM22_DU | LM22 | CN | M | sympatry | DU | Nightingale_GUT_MICRO85 | ERS5713394 (SAMEA8026177) |
| LL23_IL | LL23 | TN | M | allopatry | IL | Nightingale_GUT_MICRO41 | ERS5713350 (SAMEA8026132) |
| LL23_JE | LL23 | TN | M | allopatry | JE | Nightingale_GUT_MICRO42 | ERS5713351 (SAMEA8026133) |
| LM14_IL | LM14 | CN | M | allopatry | IL | Nightingale_GUT_MICRO68 | ERS5713377 (SAMEA8026159) |
| LM14_DU | LM14 | CN | M | allopatry | DU | Nightingale_GUT_MICRO67 | ERS5713376 (SAMEA8026158) |
| LM18_IL | LM18 | CN | M | allopatry | IL | Nightingale_GUT_MICRO74 | ERS5713383 (SAMEA8026165) |
| LM18_JE | LM18 | CN | M | allopatry | JE | Nightingale_GUT_MICRO75 | ERS5713384 (SAMEA8026166) |
| LM18_DU | LM18 | CN | M | allopatry | DU | Nightingale_GUT_MICRO73 | ERS5713382 (SAMEA8026164) |
| LM28_IL | LM28 | CN | M | sympatry | IL | Nightingale_GUT_MICRO104 | ERS5713413 (SAMEA8026196) |
| LM28_JE | LM28 | CN | M | sympatry | JE | Nightingale_GUT_MICRO105 | ERS5713414 (SAMEA8026197) |
| LL11_IL | LL11 | TN | M | sympatry | IL | Nightingale_GUT_MICRO5 | ERS5713314 (SAMEA8026096) |
| LM14_JE | LM14 | CN | M | allopatry | JE | Nightingale_GUT_MICRO69 | ERS5713378 (SAMEA8026160) |
| LL14_IL | LL14 | TN | M | sympatry | IL | Nightingale_GUT_MICRO14 | ERS5713323 (SAMEA8026105) |
| LL14_JE | LL14 | TN | M | sympatry | JE | Nightingale_GUT_MICRO15 | ERS5713324 (SAMEA8026106) |
| LL14_DU | LL14 | TN | M | sympatry | DU | Nightingale_GUT_MICRO13 | ERS5713322 (SAMEA8026104) |
| LL12_IL | LL12 | TN | M | sympatry | IL | Nightingale_GUT_MICRO8 | ERS5713317 (SAMEA8026099) |
| LL12_JE | LL12 | TN | M | sympatry | JE | Nightingale_GUT_MICRO9 | ERS5713318 (SAMEA8026100) |
| LL27_IL | LL27 | TN | M | allopatry | IL | Nightingale_GUT_MICRO50 | ERS5713359 (SAMEA8026141) |
| LL27_JE | LL27 | TN | M | allopatry | JE | Nightingale_GUT_MICRO51 | ERS5713360 (SAMEA8026142) |
| LL27_DU | LL27 | TN | M | allopatry | DU | Nightingale_GUT_MICRO49 | ERS5713358 (SAMEA8026140) |
| LM17_IL | LM17 | CN | M | allopatry | IL | Nightingale_GUT_MICRO71 | ERS5713380 (SAMEA8026162) |
| LM17_JE | LM17 | CN | M | allopatry | JE | Nightingale_GUT_MICRO72 | ERS5713381 (SAMEA8026163) |
| LM17_DU | LM17 | CN | M | allopatry | DU | Nightingale_GUT_MICRO70 | ERS5713379 (SAMEA8026161) |
| LM27_IL | LM27 | CN | M | sympatry | IL | Nightingale_GUT_MICRO101 | ERS5713410 (SAMEA8026193) |
| LM27_JE | LM27 | CN | M | sympatry | JE | Nightingale_GUT_MICRO102 | ERS5713411 (SAMEA8026194) |
| LL10_IL | LL10 | TN | M | sympatry | IL | Nightingale_GUT_MICRO2 | ERS5713311 (SAMEA8026093) |
| LL10_JE | LL10 | TN | M | sympatry | JE | Nightingale_GUT_MICRO3 | ERS5713312 (SAMEA8026094) |
| LL10_DU | LL10 | TN | M | sympatry | DU | Nightingale_GUT_MICRO1 | ERS5713310 (SAMEA8026092) |
| LL21_IL | LL21 | TN | M | allopatry | IL | Nightingale_GUT_MICRO35 | ERS5713344 (SAMEA8026126) |
| LL21_JE | LL21 | TN | M | allopatry | JE | Nightingale_GUT_MICRO36 | ERS5713345 (SAMEA8026127) |
| LL21_DU | LL21 | TN | M | allopatry | DU | Nightingale_GUT_MICRO34 | ERS5713343 (SAMEA8026125) |
| LL16_JE | LL16 | TN | M | sympatry | JE | Nightingale_GUT_MICRO21 | ERS5713330 (SAMEA8026112) |
| LL28_IL | LL28 | TN | M | allopatry | IL | Nightingale_GUT_MICRO53 | ERS5713362 (SAMEA8026144) |
| LL28_JE | LL28 | TN | M | allopatry | JE | Nightingale_GUT_MICRO54 | ERS5713363 (SAMEA8026145) |
| LL28_DU | LL28 | TN | M | allopatry | DU | Nightingale_GUT_MICRO52 | ERS5713361 (SAMEA8026143) |
| LM11_IL | LM11 | CN | M | allopatry | IL | Nightingale_GUT_MICRO59 | ERS5713368 (SAMEA8026150) |
| LM11_JE | LM11 | CN | M | allopatry | JE | Nightingale_GUT_MICRO60 | ERS5713369 (SAMEA8026151) |
| LM11_DU | LM11 | CN | M | allopatry | DU | Nightingale_GUT_MICRO58 | ERS5713367 (SAMEA8026149) |
| LM21_IL | LM21 | CN | M | sympatry | IL | Nightingale_GUT_MICRO83 | ERS5713392 (SAMEA8026175) |
| LM21_JE | LM21 | CN | M | sympatry | JE | Nightingale_GUT_MICRO84 | ERS5713393 (SAMEA8026176) |
| LM29_IL | LM29 | CN | M | sympatry | IL | Nightingale_GUT_MICRO107 | ERS5713416 (SAMEA8026199) |
| LM29_JE | LM29 | CN | M | sympatry | JE | Nightingale_GUT_MICRO108 | ERS5713417 (SAMEA8026200) |
| LM29_DU | LM29 | CN | M | sympatry | DU | Nightingale_GUT_MICRO106 | ERS5713415 (SAMEA8026198) |
| LL16_IL | LL16 | TN | M | sympatry | IL | Nightingale_GUT_MICRO20 | ERS5713329 (SAMEA8026111) |
| LL16_DU | LL16 | TN | M | sympatry | DU | Nightingale_GUT_MICRO19 | ERS5713328 (SAMEA8026110) |
| LL18_IL | LL18 | TN | M | sympatry | IL | Nightingale_GUT_MICRO26 | ERS5713335 (SAMEA8026117) |
| LL18_JE | LL18 | TN | M | sympatry | JE | Nightingale_GUT_MICRO27 | ERS5713336 (SAMEA8026118) |
| LL26_IL | LL26 | TN | M | allopatry | IL | Nightingale_GUT_MICRO47 | ERS5713356 (SAMEA8026138) |
| LL26_JE | LL26 | TN | M | allopatry | JE | Nightingale_GUT_MICRO48 | ERS5713357 (SAMEA8026139) |
| LL26_DU | LL26 | TN | M | allopatry | DU | Nightingale_GUT_MICRO46 | ERS5713355 (SAMEA8026137) |
| LM20_IL | LM20 | CN | M | allopatry | IL | Nightingale_GUT_MICRO80 | ERS5713389 (SAMEA8026172) |
| LM20_JE | LM20 | CN | M | allopatry | JE | Nightingale_GUT_MICRO81 | ERS5713390 (SAMEA8026173) |
| LM20_DU | LM20 | CN | M | allopatry | DU | Nightingale_GUT_MICRO79 | ERS5713388 (SAMEA8026171) |
| LM23_IL | LM23 | CN | M | sympatry | IL | Nightingale_GUT_MICRO89 | ERS5713398 (SAMEA8026181) |
| LM23_JE | LM23 | CN | M | sympatry | JE | Nightingale_GUT_MICRO90 | ERS5713399 (SAMEA8026182) |
| LL13_IL | LL13 | TN | M | sympatry | IL | Nightingale_GUT_MICRO11 | ERS5713320 (SAMEA8026102) |
| LL13_JE | LL13 | TN | M | sympatry | JE | Nightingale_GUT_MICRO12 | ERS5713321 (SAMEA8026103) |
| LL15_IL | LL15 | TN | M | sympatry | IL | Nightingale_GUT_MICRO17 | ERS5713326 (SAMEA8026108) |
| LL15_JE | LL15 | TN | M | sympatry | JE | Nightingale_GUT_MICRO18 | ERS5713327 (SAMEA8026109) |
| LL15_DU | LL15 | TN | M | sympatry | DU | Nightingale_GUT_MICRO16 | ERS5713325 (SAMEA8026107) |
| LL20_IL | LL20 | TN | M | allopatry | IL | Nightingale_GUT_MICRO32 | ERS5713341 (SAMEA8026123) |
| LL20_JE | LL20 | TN | M | allopatry | JE | Nightingale_GUT_MICRO33 | ERS5713342 (SAMEA8026124) |
| LM10_IL | LM10 | CN | M | allopatry | IL | Nightingale_GUT_MICRO56 | ERS5713365 (SAMEA8026147) |
| LM10_JE | LM10 | CN | M | allopatry | JE | Nightingale_GUT_MICRO57 | ERS5713366 (SAMEA8026148) |
| LM10_DU | LM10 | CN | M | allopatry | DU | Nightingale_GUT_MICRO55 | ERS5713364 (SAMEA8026146) |
| LM24_IL | LM24 | CN | M | sympatry | IL | Nightingale_GUT_MICRO92 | ERS5713401 (SAMEA8026184) |
| LM24_JE | LM24 | CN | M | sympatry | JE | Nightingale_GUT_MICRO93 | ERS5713402 (SAMEA8026185) |
| LM25_IL | LM25 | CN | M | sympatry | IL | Nightingale_GUT_MICRO95 | ERS5713404 (SAMEA8026187) |
| LM25_JE | LM25 | CN | M | sympatry | JE | Nightingale_GUT_MICRO96 | ERS5713405 (SAMEA8026188) |
| LL17_IL | LL17 | TN | M | sympatry | IL | Nightingale_GUT_MICRO23 | ERS5713332 (SAMEA8026114) |
| LL17_JE | LL17 | TN | M | sympatry | JE | Nightingale_GUT_MICRO24 | ERS5713333 (SAMEA8026115) |
| LL17_DU | LL17 | TN | M | sympatry | DU | Nightingale_GUT_MICRO22 | ERS5713331 (SAMEA8026113) |
| LL19_IL | LL19 | TN | M | allopatry | IL | Nightingale_GUT_MICRO29 | ERS5713338 (SAMEA8026120) |
| LL19_JE | LL19 | TN | M | allopatry | JE | Nightingale_GUT_MICRO30 | ERS5713339 (SAMEA8026121) |
| LL19_DU | LL19 | TN | M | allopatry | DU | Nightingale_GUT_MICRO28 | ERS5713337 (SAMEA8026119) |
| LL11_JE | LL11 | TN | M | sympatry | JE | Nightingale_GUT_MICRO6 | ERS5713315 (SAMEA8026097) |
| LL25_IL | LL25 | TN | M | allopatry | IL | Nightingale_GUT_MICRO44 | ERS5713353 (SAMEA8026135) |
| LL25_JE | LL25 | TN | M | allopatry | JE | Nightingale_GUT_MICRO45 | ERS5713354 (SAMEA8026136) |
| LL25_DU | LL25 | TN | M | allopatry | DU | Nightingale_GUT_MICRO43 | ERS5713352 (SAMEA8026134) |
| LM12_IL | LM12 | CN | M | allopatry | IL | Nightingale_GUT_MICRO62 | ERS5713371 (SAMEA8026153) |
| LM12_JE | LM12 | CN | M | allopatry | JE | Nightingale_GUT_MICRO63 | ERS5713372 (SAMEA8026154) |
| LM12_DU | LM12 | CN | M | allopatry | DU | Nightingale_GUT_MICRO61 | ERS5713370 (SAMEA8026152) |
| LM13_IL | LM13 | CN | M | allopatry | IL | Nightingale_GUT_MICRO65 | ERS5713374 (SAMEA8026156) |
| LM13_JE | LM13 | CN | M | allopatry | JE | Nightingale_GUT_MICRO66 | ERS5713375 (SAMEA8026157) |
| LL23_DU | LL23 | TN | M | allopatry | DU | Nightingale_GUT_MICRO40 | ERS5713349 (SAMEA8026131) |
| LM28_DU | LM28 | CN | M | sympatry | DU | Nightingale_GUT_MICRO103 | ERS5713412 (SAMEA8026195) |
| LL12_DU | LL12 | TN | M | sympatry | DU | Nightingale_GUT_MICRO7 | ERS5713316 (SAMEA8026098) |
| LM27_DU | LM27 | CN | M | sympatry | DU | Nightingale_GUT_MICRO100 | ERS5713409 (SAMEA8026192) |
| LL13_DU | LL13 | TN | M | sympatry | DU | Nightingale_GUT_MICRO10 | ERS5713319 (SAMEA8026101) |
| LM21_DU | LM21 | CN | M | sympatry | DU | Nightingale_GUT_MICRO82 | ERS5713391 (SAMEA8026174) |
| LL18_DU | LL18 | TN | M | sympatry | DU | Nightingale_GUT_MICRO25 | ERS5713334 (SAMEA8026116) |
| LM23_DU | LM23 | CN | M | sympatry | DU | Nightingale_GUT_MICRO88 | ERS5713397 (SAMEA8026180) |
| LL20_DU | LL20 | TN | M | allopatry | DU | Nightingale_GUT_MICRO31 | ERS5713340 (SAMEA8026122) |
| LM25_DU | LM25 | CN | M | sympatry | DU | Nightingale_GUT_MICRO94 | ERS5713403 (SAMEA8026186) |
| LM24_DU | LM24 | CN | M | sympatry | DU | Nightingale_GUT_MICRO91 | ERS5713400 (SAMEA8026183) |
| LM13_DU | LM13 | CN | M | allopatry | DU | Nightingale_GUT_MICRO64 | ERS5713373 (SAMEA8026155) |
| LM26_IL | LM26 | CN | M | sympatry | IL | Nightingale_GUT_MICRO98 | ERS5713407 (SAMEA8026190) |
| LL22_IL | LL22 | TN | M | allopatry | IL | Nightingale_GUT_MICRO38 | ERS5713347 (SAMEA8026129) |
| LM26_JE | LM26 | CN | M | sympatry | JE | Nightingale_GUT_MICRO99 | ERS5713408 (SAMEA8026191) |
| LL22_JE | LL22 | TN | M | allopatry | JE | Nightingale_GUT_MICRO39 | ERS5713348 (SAMEA8026130) |
| LM26_DU | LM26 | CN | M | sympatry | DU | Nightingale_GUT_MICRO97 | ERS5713406 (SAMEA8026189) |
| LL11_DU | LL11 | TN | M | sympatry | DU | Nightingale_GUT_MICRO4 | ERS5713313 (SAMEA8026095) |
| LL22_DU | LL22 | TN | M | allopatry | DU | Nightingale_GUT_MICRO37 | ERS5713346 (SAMEA8026128) |
| LM19_IL | LM19 | CN | M | allopatry | IL | Nightingale_GUT_MICRO77 | ERS5713386 (SAMEA8026169) |
| LM19_JE | LM19 | CN | M | allopatry | JE | Nightingale_GUT_MICRO78 | ERS5713387 (SAMEA8026170) |
| LM19_DU | LM19 | CN | M | allopatry | DU | Nightingale_GUT_MICRO76 | ERS5713385 (SAMEA8026167) |
| LM22_IL | LM22 | CN | M | sympatry | IL | Nightingale_GUT_MICRO86 | ERS5713395 (SAMEA8026178) |
| LM22_JE | LM22 | CN | M | sympatry | JE | Nightingale_GUT_MICRO87 | ERS5713396 (SAMEA8026179) |
| LM22_DU | LM22 | CN | M | sympatry | DU | Nightingale_GUT_MICRO85 | ERS5713394 (SAMEA8026177) |
| LL23_IL | LL23 | TN | M | allopatry | IL | Nightingale_GUT_MICRO41 | ERS5713350 (SAMEA8026132) |
| LL23_JE | LL23 | TN | M | allopatry | JE | Nightingale_GUT_MICRO42 | ERS5713351 (SAMEA8026133) |
| LM14_IL | LM14 | CN | M | allopatry | IL | Nightingale_GUT_MICRO68 | ERS5713377 (SAMEA8026159) |
| LM14_DU | LM14 | CN | M | allopatry | DU | Nightingale_GUT_MICRO67 | ERS5713376 (SAMEA8026158) |
| LM18_IL | LM18 | CN | M | allopatry | IL | Nightingale_GUT_MICRO74 | ERS5713383 (SAMEA8026165) |
| LM18_JE | LM18 | CN | M | allopatry | JE | Nightingale_GUT_MICRO75 | ERS5713384 (SAMEA8026166) |
| LM18_DU | LM18 | CN | M | allopatry | DU | Nightingale_GUT_MICRO73 | ERS5713382 (SAMEA8026164) |
| LM28_IL | LM28 | CN | M | sympatry | IL | Nightingale_GUT_MICRO104 | ERS5713413 (SAMEA8026196) |
| LM28_JE | LM28 | CN | M | sympatry | JE | Nightingale_GUT_MICRO105 | ERS5713414 (SAMEA8026197) |
| LL11_IL | LL11 | TN | M | sympatry | IL | Nightingale_GUT_MICRO5 | ERS5713314 (SAMEA8026096) |
| LM14_JE | LM14 | CN | M | allopatry | JE | Nightingale_GUT_MICRO69 | ERS5713378 (SAMEA8026160) |
| LL14_IL | LL14 | TN | M | sympatry | IL | Nightingale_GUT_MICRO14 | ERS5713323 (SAMEA8026105) |
| LL14_JE | LL14 | TN | M | sympatry | JE | Nightingale_GUT_MICRO15 | ERS5713324 (SAMEA8026106) |
| LL14_DU | LL14 | TN | M | sympatry | DU | Nightingale_GUT_MICRO13 | ERS5713322 (SAMEA8026104) |
| LL12_IL | LL12 | TN | M | sympatry | IL | Nightingale_GUT_MICRO8 | ERS5713317 (SAMEA8026099) |
| LL12_JE | LL12 | TN | M | sympatry | JE | Nightingale_GUT_MICRO9 | ERS5713318 (SAMEA8026100) |
| LL27_IL | LL27 | TN | M | allopatry | IL | Nightingale_GUT_MICRO50 | ERS5713359 (SAMEA8026141) |
| LL27_JE | LL27 | TN | M | allopatry | JE | Nightingale_GUT_MICRO51 | ERS5713360 (SAMEA8026142) |
| LL27_DU | LL27 | TN | M | allopatry | DU | Nightingale_GUT_MICRO49 | ERS5713358 (SAMEA8026140) |
| LM17_IL | LM17 | CN | M | allopatry | IL | Nightingale_GUT_MICRO71 | ERS5713380 (SAMEA8026162) |
| LM17_JE | LM17 | CN | M | allopatry | JE | Nightingale_GUT_MICRO72 | ERS5713381 (SAMEA8026163) |
| LM17_DU | LM17 | CN | M | allopatry | DU | Nightingale_GUT_MICRO70 | ERS5713379 (SAMEA8026161) |
| LM27_IL | LM27 | CN | M | sympatry | IL | Nightingale_GUT_MICRO101 | ERS5713410 (SAMEA8026193) |
| LM27_JE | LM27 | CN | M | sympatry | JE | Nightingale_GUT_MICRO102 | ERS5713411 (SAMEA8026194) |
| LL10_IL | LL10 | TN | M | sympatry | IL | Nightingale_GUT_MICRO2 | ERS5713311 (SAMEA8026093) |
| LL10_JE | LL10 | TN | M | sympatry | JE | Nightingale_GUT_MICRO3 | ERS5713312 (SAMEA8026094) |
| LL10_DU | LL10 | TN | M | sympatry | DU | Nightingale_GUT_MICRO1 | ERS5713310 (SAMEA8026092) |
| LL21_IL | LL21 | TN | M | allopatry | IL | Nightingale_GUT_MICRO35 | ERS5713344 (SAMEA8026126) |
| LL21_JE | LL21 | TN | M | allopatry | JE | Nightingale_GUT_MICRO36 | ERS5713345 (SAMEA8026127) |
| LL21_DU | LL21 | TN | M | allopatry | DU | Nightingale_GUT_MICRO34 | ERS5713343 (SAMEA8026125) |
| LL16_JE | LL16 | TN | M | sympatry | JE | Nightingale_GUT_MICRO21 | ERS5713330 (SAMEA8026112) |
| LL28_IL | LL28 | TN | M | allopatry | IL | Nightingale_GUT_MICRO53 | ERS5713362 (SAMEA8026144) |
| LL28_JE | LL28 | TN | M | allopatry | JE | Nightingale_GUT_MICRO54 | ERS5713363 (SAMEA8026145) |
| LL28_DU | LL28 | TN | M | allopatry | DU | Nightingale_GUT_MICRO52 | ERS5713361 (SAMEA8026143) |
| LM11_IL | LM11 | CN | M | allopatry | IL | Nightingale_GUT_MICRO59 | ERS5713368 (SAMEA8026150) |
| LM11_JE | LM11 | CN | M | allopatry | JE | Nightingale_GUT_MICRO60 | ERS5713369 (SAMEA8026151) |
| LM11_DU | LM11 | CN | M | allopatry | DU | Nightingale_GUT_MICRO58 | ERS5713367 (SAMEA8026149) |
| LM21_IL | LM21 | CN | M | sympatry | IL | Nightingale_GUT_MICRO83 | ERS5713392 (SAMEA8026175) |
| LM21_JE | LM21 | CN | M | sympatry | JE | Nightingale_GUT_MICRO84 | ERS5713393 (SAMEA8026176) |
| LM29_IL | LM29 | CN | M | sympatry | IL | Nightingale_GUT_MICRO107 | ERS5713416 (SAMEA8026199) |
| LM29_JE | LM29 | CN | M | sympatry | JE | Nightingale_GUT_MICRO108 | ERS5713417 (SAMEA8026200) |
| LM29_DU | LM29 | CN | M | sympatry | DU | Nightingale_GUT_MICRO106 | ERS5713415 (SAMEA8026198) |
| LL16_IL | LL16 | TN | M | sympatry | IL | Nightingale_GUT_MICRO20 | ERS5713329 (SAMEA8026111) |
| LL16_DU | LL16 | TN | M | sympatry | DU | Nightingale_GUT_MICRO19 | ERS5713328 (SAMEA8026110) |
| LL18_IL | LL18 | TN | M | sympatry | IL | Nightingale_GUT_MICRO26 | ERS5713335 (SAMEA8026117) |
| LL18_JE | LL18 | TN | M | sympatry | JE | Nightingale_GUT_MICRO27 | ERS5713336 (SAMEA8026118) |
| LL26_IL | LL26 | TN | M | allopatry | IL | Nightingale_GUT_MICRO47 | ERS5713356 (SAMEA8026138) |
| LL26_JE | LL26 | TN | M | allopatry | JE | Nightingale_GUT_MICRO48 | ERS5713357 (SAMEA8026139) |
| LL26_DU | LL26 | TN | M | allopatry | DU | Nightingale_GUT_MICRO46 | ERS5713355 (SAMEA8026137) |
| LM20_IL | LM20 | CN | M | allopatry | IL | Nightingale_GUT_MICRO80 | ERS5713389 (SAMEA8026172) |
| LM20_JE | LM20 | CN | M | allopatry | JE | Nightingale_GUT_MICRO81 | ERS5713390 (SAMEA8026173) |
| LM20_DU | LM20 | CN | M | allopatry | DU | Nightingale_GUT_MICRO79 | ERS5713388 (SAMEA8026171) |
| LM23_IL | LM23 | CN | M | sympatry | IL | Nightingale_GUT_MICRO89 | ERS5713398 (SAMEA8026181) |
| LM23_JE | LM23 | CN | M | sympatry | JE | Nightingale_GUT_MICRO90 | ERS5713399 (SAMEA8026182) |
| LL13_IL | LL13 | TN | M | sympatry | IL | Nightingale_GUT_MICRO11 | ERS5713320 (SAMEA8026102) |
| LL13_JE | LL13 | TN | M | sympatry | JE | Nightingale_GUT_MICRO12 | ERS5713321 (SAMEA8026103) |
| LL15_IL | LL15 | TN | M | sympatry | IL | Nightingale_GUT_MICRO17 | ERS5713326 (SAMEA8026108) |
| LL15_JE | LL15 | TN | M | sympatry | JE | Nightingale_GUT_MICRO18 | ERS5713327 (SAMEA8026109) |
| LL15_DU | LL15 | TN | M | sympatry | DU | Nightingale_GUT_MICRO16 | ERS5713325 (SAMEA8026107) |
| LL20_IL | LL20 | TN | M | allopatry | IL | Nightingale_GUT_MICRO32 | ERS5713341 (SAMEA8026123) |
| LL20_JE | LL20 | TN | M | allopatry | JE | Nightingale_GUT_MICRO33 | ERS5713342 (SAMEA8026124) |
| LM10_IL | LM10 | CN | M | allopatry | IL | Nightingale_GUT_MICRO56 | ERS5713365 (SAMEA8026147) |
| LM10_JE | LM10 | CN | M | allopatry | JE | Nightingale_GUT_MICRO57 | ERS5713366 (SAMEA8026148) |
| LM10_DU | LM10 | CN | M | allopatry | DU | Nightingale_GUT_MICRO55 | ERS5713364 (SAMEA8026146) |
| LM24_IL | LM24 | CN | M | sympatry | IL | Nightingale_GUT_MICRO92 | ERS5713401 (SAMEA8026184) |
| LM24_JE | LM24 | CN | M | sympatry | JE | Nightingale_GUT_MICRO93 | ERS5713402 (SAMEA8026185) |
| LM25_IL | LM25 | CN | M | sympatry | IL | Nightingale_GUT_MICRO95 | ERS5713404 (SAMEA8026187) |
| LM25_JE | LM25 | CN | M | sympatry | JE | Nightingale_GUT_MICRO96 | ERS5713405 (SAMEA8026188) |
| LL17_IL | LL17 | TN | M | sympatry | IL | Nightingale_GUT_MICRO23 | ERS5713332 (SAMEA8026114) |
| LL17_JE | LL17 | TN | M | sympatry | JE | Nightingale_GUT_MICRO24 | ERS5713333 (SAMEA8026115) |
| LL17_DU | LL17 | TN | M | sympatry | DU | Nightingale_GUT_MICRO22 | ERS5713331 (SAMEA8026113) |
| LL19_IL | LL19 | TN | M | allopatry | IL | Nightingale_GUT_MICRO29 | ERS5713338 (SAMEA8026120) |
| LL19_JE | LL19 | TN | M | allopatry | JE | Nightingale_GUT_MICRO30 | ERS5713339 (SAMEA8026121) |
| LL19_DU | LL19 | TN | M | allopatry | DU | Nightingale_GUT_MICRO28 | ERS5713337 (SAMEA8026119) |
| LL11_JE | LL11 | TN | M | sympatry | JE | Nightingale_GUT_MICRO6 | ERS5713315 (SAMEA8026097) |
| LL25_IL | LL25 | TN | M | allopatry | IL | Nightingale_GUT_MICRO44 | ERS5713353 (SAMEA8026135) |
| LL25_JE | LL25 | TN | M | allopatry | JE | Nightingale_GUT_MICRO45 | ERS5713354 (SAMEA8026136) |
| LL25_DU | LL25 | TN | M | allopatry | DU | Nightingale_GUT_MICRO43 | ERS5713352 (SAMEA8026134) |
| LM12_IL | LM12 | CN | M | allopatry | IL | Nightingale_GUT_MICRO62 | ERS5713371 (SAMEA8026153) |
| LM12_JE | LM12 | CN | M | allopatry | JE | Nightingale_GUT_MICRO63 | ERS5713372 (SAMEA8026154) |
| LM12_DU | LM12 | CN | M | allopatry | DU | Nightingale_GUT_MICRO61 | ERS5713370 (SAMEA8026152) |
| LM13_IL | LM13 | CN | M | allopatry | IL | Nightingale_GUT_MICRO65 | ERS5713374 (SAMEA8026156) |
| LM13_JE | LM13 | CN | M | allopatry | JE | Nightingale_GUT_MICRO66 | ERS5713375 (SAMEA8026157) |
| LL23_DU | LL23 | TN | M | allopatry | DU | Nightingale_GUT_MICRO40 | ERS5713349 (SAMEA8026131) |
| LM28_DU | LM28 | CN | M | sympatry | DU | Nightingale_GUT_MICRO103 | ERS5713412 (SAMEA8026195) |
| LL12_DU | LL12 | TN | M | sympatry | DU | Nightingale_GUT_MICRO7 | ERS5713316 (SAMEA8026098) |
| LM27_DU | LM27 | CN | M | sympatry | DU | Nightingale_GUT_MICRO100 | ERS5713409 (SAMEA8026192) |
| LL13_DU | LL13 | TN | M | sympatry | DU | Nightingale_GUT_MICRO10 | ERS5713319 (SAMEA8026101) |
| LM21_DU | LM21 | CN | M | sympatry | DU | Nightingale_GUT_MICRO82 | ERS5713391 (SAMEA8026174) |
| LL18_DU | LL18 | TN | M | sympatry | DU | Nightingale_GUT_MICRO25 | ERS5713334 (SAMEA8026116) |
| LM23_DU | LM23 | CN | M | sympatry | DU | Nightingale_GUT_MICRO88 | ERS5713397 (SAMEA8026180) |
| LL20_DU | LL20 | TN | M | allopatry | DU | Nightingale_GUT_MICRO31 | ERS5713340 (SAMEA8026122) |
| LM25_DU | LM25 | CN | M | sympatry | DU | Nightingale_GUT_MICRO94 | ERS5713403 (SAMEA8026186) |
| LM24_DU | LM24 | CN | M | sympatry | DU | Nightingale_GUT_MICRO91 | ERS5713400 (SAMEA8026183) |
| LM13_DU | LM13 | CN | M | allopatry | DU | Nightingale_GUT_MICRO64 | ERS5713373 (SAMEA8026155) |
| LM26_IL | LM26 | CN | M | sympatry | IL | Nightingale_GUT_MICRO98 | ERS5713407 (SAMEA8026190) |
| LL22_IL | LL22 | TN | M | allopatry | IL | Nightingale_GUT_MICRO38 | ERS5713347 (SAMEA8026129) |
| LM26_JE | LM26 | CN | M | sympatry | JE | Nightingale_GUT_MICRO99 | ERS5713408 (SAMEA8026191) |
| LL22_JE | LL22 | TN | M | allopatry | JE | Nightingale_GUT_MICRO39 | ERS5713348 (SAMEA8026130) |
| LM26_DU | LM26 | CN | M | sympatry | DU | Nightingale_GUT_MICRO97 | ERS5713406 (SAMEA8026189) |
| LL11_DU | LL11 | TN | M | sympatry | DU | Nightingale_GUT_MICRO4 | ERS5713313 (SAMEA8026095) |
| LL22_DU | LL22 | TN | M | allopatry | DU | Nightingale_GUT_MICRO37 | ERS5713346 (SAMEA8026128) |
